# Modelling the impact of tailored behavioural interventions on chlamydia transmission

**DOI:** 10.1038/s41598-021-81675-w

**Published:** 2021-01-25

**Authors:** Daphne A. van Wees, Chantal den Daas, Mirjam E. E. Kretzschmar, Janneke C. M. Heijne

**Affiliations:** 1 Center for Infectious Disease Control, National Institute for Public Health and the Environment (RIVM), P.O. Box 1, 3720 BA Bilthoven, The Netherlands; 2 Department of Interdisciplinary Social Science, Faculty of Social and Behavioural Sciences, Utrecht University, Utrecht, The Netherlands; 3 Julius Center for Health Sciences and Primary Care, University Medical Center Utrecht, Utrecht University, Utrecht, The Netherlands

**Keywords:** Infectious diseases, Human behaviour, Computational models

## Abstract

Behavioural interventions tailored to psychological characteristics of an individual can effectively achieve risk-reducing behaviour. The impact of tailored interventions on population-level chlamydia prevalence is unknown. We aimed to assess the impact on overall chlamydia prevalence five years after the introduction of an intervention aimed at increasing self-efficacy, social norms, attitudes and intentions towards condom use (i.e., condom intervention), and an intervention aimed at increasing health goals and decreasing impulsiveness (i.e., impulsiveness intervention). A pair model, informed by longitudinal psychological and behavioural data of young heterosexuals visiting sexual health centers, with susceptible-infected-susceptible structure was developed. The intervention effect was defined as an increased proportion of each subgroup moving to the desired subgroup (i.e., lower risk subgroup). Interventions tailored to subgroup-specific characteristics, assuming differential intervention effects in each subgroup, more effectively reduced overall chlamydia prevalence compared to non-tailored interventions. The most effective intervention was the tailored condom intervention, which was assumed to result in a relative reduction in chlamydia prevalence of 18% versus 12% in the non-tailored scenario. Thus, it is important to assess multiple psychological and behavioural characteristics of individuals. Tailored interventions may be more successful in achieving risk-reducing behaviour, and consequently, reduce chlamydia prevalence more effectively.

## Introduction

Sexually transmitted infections (STI) are among the most common causes of morbidity worldwide^1,2^, and control of STI is proven to be challenging^1,3,4^. Psychological mechanisms of behaviour, such as risk perception, knowledge, or attitudes regarding condom use and STI testing^5–8^, may play an important role in improving STI control^9,10^. For example, psychological characteristics may also help to identify different types of individuals in a population at high risk for acquiring chlamydia (i.e., many partners and inconsistent condom use) that would not be identified based on sexual behaviour alone^11,12^. Previous studies showed that high impulsiveness ^13–15^, low perceived importance of health^8^, and STI-related stigma and shame^16–19^, are associated with risky decision-making in terms of sexual behaviour. Taking differences between individuals in psychological and behavioural characteristics into account in behavioural interventions may change sexual behaviour and reduce STI prevalence more effectively.

A good context to study the impact of behavioural interventions on prevalence is the bacterial STI *Chlamydia trachomatis* (chlamydia). Chlamydia remains the most commonly diagnosed bacterial STI among young heterosexuals with more than 130 million new cases per year worldwide^1,2^. Chlamydia infections can lead to serious reproductive complications in females, such as pelvic inflammatory disease (PID), ectopic pregnancy, and tubal factor infertility ^20–23^, which is of public health importance. Even though many Western countries have chlamydia control and prevention efforts in place, such as sexual health promotion and education^24^, or national screening programs^25–30^, chlamydia prevalence has not declined over the past years, and the reasons for this remain unclear. Previous studies have shown that targeting multiple psychological characteristics, such as decreasing impulsive behaviour and increasing the perceived importance of health^8^, or improving self-efficacy, social norms and attitudes regarding condom use^31^, may achieve risk-reducing behaviour. However, few studies have investigated the impact of behavioural interventions on chlamydia prevalence^32,33^.

Behavioural interventions are often STI clinic-based^32,34^, as individuals visiting the STI clinic at sexual health centers (SHC) tend to engage in higher risk sexual behaviour than the general population^35,36^, and potentially benefit the most from behavioural interventions^34^. It was previously shown that individuals who were diagnosed with a chlamydia infection engaged in more risk-reducing sexual behaviour after receiving the test results, whereas individuals who tested chlamydia negative did not change their sexual behaviour or even engaged in behaviours related to increased risk (i.e., decreased condom use) after testing^9,10,37^. Since all STI clinic visitors are viewed to be at high risk for chlamydia based on their sexual behaviour^35^, individuals who tested chlamydia negative remain at increased risk of acquiring chlamydia in the future when they do not change their behaviour in a more risk-reducing direction. Thus, identifying behavioural interventions that can achieve risk-reducing behaviour more effectively might be needed to reduce chlamydia prevalence, especially for individuals testing chlamydia negative.

To assess the impact of behavioural interventions on behaviour and chlamydia prevalence, mathematical modelling could be used^33^. In most mathematical models describing chlamydia transmission, the population is divided into groups with different levels of chlamydia risk to account for heterogeneity in sexual behaviour^38^, but psychological characteristics are rarely incorporated. Furthermore, previous modelling studies that examined the impact of psychological characteristics on behaviour and infectious disease transmission dynamics incorporated only a single characteristic in the model (i.e., risk perception, or beliefs)^39–41^. However, behavioural interventions usually target multiple characteristics at the same time to achieve the desired behaviour^32^. Incorporating multiple psychological characteristics into mathematical models, in addition to sexual behaviour, could enable a more realistic assessment of the impact of interventions tailored to individual- or subgroup-specific characteristics on chlamydia prevalence^42^. Furthermore, to be able to estimate the effect of behavioural interventions on these psychological characteristics, incorporating behaviour change into the model is necessary. Previous studies modelling transmission dynamics of human immunodeficiency virus (HIV) found that models that do not incorporate behaviour change might underestimate the impact of interventions on disease prevalence^43–45^. To our knowledge, a model incorporating multiple psychological characteristics and behaviour change to explore the impact of interventions on chlamydia transmission is not yet developed.

In this study, we incorporated subgroups based on multiple psychological and behavioural characteristics, and behaviour change, among young heterosexuals into a mathematical model. First, we aimed to assess the impact of different behavioural interventions tailored to the characteristics of these subgroups on overall chlamydia prevalence compared to interventions that were not tailored to subgroup-specific characteristics. Second, we aimed to compare the impact of tailored behavioural interventions targeted only to individuals who were diagnosed with chlamydia, or only to individuals who tested chlamydia negative.

## Methods

### Data

Data from a longitudinal cohort study among heterosexual males and females aged 18–24 years visiting sexual health centers (SHC) in the Netherlands was used to parameterize the model. Details of this study, called ‘Mathematical models incorporating Psychological determinants: control of Chlamydia Transmission’ (iMPaCT), can be found elsewhere^46^. In short, participants were recruited at the SHC in Amsterdam, Kennemerland, Hollands-Noorden, and Twente between November 2016 and July 2018. Participants filled out an online questionnaire assessing psychological and behavioural characteristics at four different time points: baseline, 3-week, 6-month, and 1-year follow-up, and all participants were tested for chlamydia at baseline with nucleic acid amplification tests (NAAT). The iMPaCT study was performed in accordance with relevant guidelines and regulations, and approved by the Medical Ethical Committee of the University Medical Center Utrecht, the Netherlands (NL57481.094.16/METC18-363/D/Dutch Trial Register NTR-6307). Informed consent was obtained from all participants.

### Description of the model

A pair compartmental model with a susceptible-infected-susceptible structure (SIS) representing heterosexuals aged 18–24 years was developed, based on previous work^42^. In short, people can either be susceptible or infected with chlamydia, and become infected with a transmission probability per condomless sex act in a pair of a susceptible and an infected individual. Infected individuals can become susceptible again by naturally clearing the infection, or after testing and treatment. The duration of asymptomatic infection was informed by the literature^47,48^. People enter the model as susceptible singles at age 18 (i.e., influx) and leave the model at age 25 (i.e., aging out). Partnership duration was incorporated explicitly: single females and males can form pairs and break up at any point in time.

The model was extended in three ways: by incorporating concurrency, subgroups of individuals characterized by multiple psychological characteristics and behaviour change (Supplementary Material, text S1). Concurrency was incorporated as having one-time sexual encounters or ‘one-night stands’ for people in partnerships^49^. Furthermore, people in a single state were able to have one-night stands as well (Supplementary Material, text S1.4.2 and S1.4.3).

### Subgroups

We incorporated four subgroups based on a combination of different behavioural and psychological characteristics that were identified using the iMPaCT data and latent transition analyses (LTA)^50^. The entropy value in the LTA was very high (> 0.8, indicating good classification), which means that the risk of misclassification in the subgroups was very limited. The identified subgroups were labelled based on the most distinctive psychological and behavioural characteristics (Supplementary Material, table S1). Two subgroups were characterized by behaviour associated with higher chlamydia risk (e.g., high number of partners, inconsistent condom use), compared to the other subgroups. One of these higher risk subgroups reporting significantly lower self-esteem, and self-efficacy, and high impulsiveness, and was referred to as the ‘insecure’ subgroup. The other higher risk subgroup reporting higher self-esteem, less positive attitudes and lower intentions towards condom use and STI testing was referred to as the ‘confident’ subgroup. The two other subgroups were characterized by behaviour associated with lower chlamydia risk (e.g., low number of partners, more consistent condom use). The lower risk subgroup reporting significantly lower impulsiveness was referred to as the ‘low-impulsivity’ subgroup. Participants in the subgroup reporting more consistent condom use compared to the other subgroups were referred to as the ‘condom-using’ subgroup.

A mixing parameter, ranging from fully assortative (only like-with-like mixing) to fully proportionate mixing (random mixing) between subgroups, was incorporated in the model to determine mixing between individuals of the four subgroups (Supplementary Material, text S1.3). To inform the behavioural parameters of the model, we used, the median number of sex acts per week, condom use, number of partners per year, number of one-night stands per year, and percentage of the population in a partnership in each subgroup in the iMPaCT data (see Table 1, and Supplementary Material, table S2 and text S1.4). Parameters were assumed to be the same in males and females, because the number of male participants in the iMPaCT study was too low to stratify for gender. We further assumed that behaviour in partnerships was equally determined by individuals from different subgroups, meaning that in pairs of individuals from different subgroups, the mean of the parameter values (i.e., number of sex acts, condom use, partnership duration) of each subgroup was taken.

**Table 1:** Subgroup characteristics among heterosexual males and females aged 18–24 years in the iMPaCT study (n = 810) to inform the mathematical model.

iMPaCT data^a^	Low-impulsivity subgroup		Condom-using subgroup		Insecure subgroup		Confident subgroup	
	%	95% CI	%	95% CI	%	95% CI	%	95% CI
Class size, baseline	10	8.1–12.3	38	34.7–41.4	21	18.3–23.9	31	27.9–34.3
Class size, one-year follow-up	30	26.9–33.2	28	25.0–31.2	17	14.6–19.8	25	22.1–28.0
Chlamydia positivity rate, baseline	7	2.9–14.7	14	10.3–17.9	12	7.9–17.9	15	11.1–19.9
Percentage of population in a partnership^b^	68	55.6–77.8	54	47.4–59.5	61	52.4–69.6	64	57.2–70.9

	Median		Median		Median		Median	
Number of sex acts, past 4 weeks	5		3		4		4	
Percentage condom use in general^c^	25		75		25		25	
Number of partners, per year^b^	2		3		3		4	
Number of one-night stands, per year^b^	0		1		1		2	
Percentage of one-night stands, single^b^	67		77		76		71	
Percentage of one-night stands, in a pair^b^	33		23		24		29	
Duration of most recent partnership, in days^d^	139 (IQR 55–599)		73 (IQR 30-196)		51 (IQR 16-208)		58 (IQR 15-168)	

^a^Calculated using behavioural data from the iMPaCT study (see electronic supplementary material, S1.4).

^b^Subset of the study population (n = 627), excluding participants who reported overlap of two most recent partnerships (when duration partnership ≥ 1 day, i.e., not a one-night stand).

^c^Condom use in general ‘How often do you use condoms in general?’ on a scale from 0% (never) to 100% (always).

^d^Subset of the study population (n = 388), excluding participants who reported overlap of two most recent partnerships (when duration partnership ≥ 1 day) or whose most recent partnership was a one-night stand (when duration most recent partnership ≤ 1 day).

### Behaviour change

Behaviour change was incorporated in the model by (1) allowing individuals to move to another subgroup after testing, (2) allowing individuals to move to another subgroup independent of testing. The probability of moving to another subgroup (i.e., transition probability) was dependent on the subgroup. The transition probabilities in the model were informed by the movement between subgroups over time in the iMPaCT data and latent transition analysis (Supplementary Material, table S3)^50^. Transition probabilities after a chlamydia positive test result were different than the transition probabilities after a chlamydia negative test result. These probabilities were informed by the proportion of iMPaCT participants moving from a subgroup at baseline to another subgroup at three-week follow-up. Transition probabilities for behaviour change independent of testing were informed by the proportion of participants in each subgroup moving from a subgroup at baseline to another subgroup at one-year follow-up. We calculated the transition probabilities relative to baseline, because at baseline the participants have not yet attended the SHC, whereas at three-week or six-month follow-up the transition rates may be affected by the SHC visit and chlamydia test results. For both types of behaviour change (i.e., after testing, or behaviour change without testing), after moving to another subgroup, individuals adopt the characteristics of the subgroup they moved to in terms of behavioural parameters, and transition probabilities to all other subgroups (Supplementary Material, text S1.2, figure S2, table S2, and table S3). This means that individuals can also move back to the subgroup they initially started in.

### Model fitting

The transmission probability per sex act, and testing uptake in each subgroup were unknown factors, and were calibrated to the overall and subgroup specific chlamydia positivity rates found in the iMPaCT study (Table 1). Calibration was done using the L-BFGS-B method in the optim-function R version 3.6.0^51^, which provides algorithms for general-purpose optimizations^52^. Furthermore, the influx rate of susceptible individuals was fitted such that the steady state proportions of the model population in each subgroup were similar to the subgroup proportions at one-year follow-up in the iMPaCT data (Table 1).

### Interventions

The impact of three behavioural intervention scenarios were explored in the model: condom promotion at SHC, an impulsiveness-reducing intervention at SHC, and a condom promotion campaign. The aims of condom promotion at SHC and in the condom promotion campaign in the model were increasing self-efficacy, social norms, attitudes and intentions towards condom use in order to increase condom use, based on effects of a real-life Dutch condom promotion campaign^31^. The aim of the impulsiveness-reducing intervention was increasing health goals and decreasing impulsive behaviour in order to decrease risky decision-making (i.e., decreased number of partners, increased condom use), based on a psychological experimental study^8^. The direction of change in specific psychological characteristics in the behavioural intervention was based on the aforementioned studies^8,31^, but the effect size in the model was assumed, based on expert opinions, and changes over time in psychological characteristics and sexual behaviour (1%-20% change expressed in percentage points) in the literature^9,37^ (Supplementary Material, table S5).

Self-efficacy, social norms, attitudes and intentions towards condom use were highest in the condom-using subgroup. Therefore, it was assumed that after introduction of the condom promotion at SHC and in the condom promotion campaign, the transition probability for individuals from the other subgroups to move to the condom-using subgroup in the model increased (Supplementary Material, text S2.1). Similarly, as impulsiveness was lowest in the low-impulsivity subgroup, we assumed that the transition probability for individuals from the other subgroups to move to the low-impulsivity subgroup increased after the introduction of the impulsiveness intervention (Supplementary Material, table S4).

### Intervention effect

The intervention effect was defined as the relative difference between the original transition probabilities per year and the intervention transition probabilities per year. First, we assumed that the intervention effect was the same in each subgroup (i.e., non-differential intervention effect), meaning that the interventions were not tailored to subgroup-specific characteristics. Second, we assumed that the intervention effect was different in each subgroup (i.e., differential intervention effect) (Supplementary Table S5), meaning that the interventions were tailored to subgroup-specific characteristics. For example, the intervention effect of the impulsiveness-reducing intervention was assumed to be larger in the insecure subgroup, because this subgroup was characterized by higher impulsivity compared to the other subgroups. The condom promotion campaign was targeted at everyone in the model, and was not dependent on testing (i.e., changed transition probabilities without testing per year).

### Outcomes

The impact of the intervention scenarios was defined as the relative reduction in overall chlamydia prevalence between baseline (before the introduction of the intervention) and five years after the introduction of the intervention (Supplementary text S2.2). First, we compared the impact of assuming non-differential versus differential intervention effects on overall chlamydia prevalence of the condom promotion at SHC, impulsiveness intervention at SHC, and the condom promotion campaign separately. Second, we compared the impact of assuming differential intervention effects between the three behavioural interventions. Last, we compared the impact of targeting the condom promotion at SHC, and the impulsiveness intervention at SHC, only to individuals who were diagnosed with (CT+), and only to individuals who tested chlamydia negative (CT−), on overall chlamydia prevalence.

To assess the impact of differential intervention effects on subgroup-specific chlamydia prevalence, the relative reduction in chlamydia prevalence was calculated for each subgroup separately. The relative reduction after the condom promotion and impulsiveness intervention at SHC, targeting only CT+, only CT−, or all tested individuals, was calculated for each subgroup. Furthermore, the impact of the condom promotion campaign on subgroup-specific prevalence was assessed.

### Uncertainty analyses

We performed uncertainty analyses on the impact of tailored interventions by changing the mixing parameter value from zero (fully assortative mixing) to one (fully proportionate mixing). This was done by recalibrating the transmission probability to the steady-state prevalence, and keeping all other parameter values fixed at the baseline values. As some of the characteristics targeted in the interventions were relatively similar in the insecure and confident subgroup, and in the low-impulsivity and condom-using subgroup, we also tested the uncertainty of the assumed differential intervention effects (Supplementary Material, table S5). We modelled partially differential intervention effects, which means that the intervention effect was assumed to be of equal size in the low-impulsivity/condom-using subgroup and in the insecure/confident subgroup. Furthermore, we modelled the differential intervention effects again, but with an alternate effect for the insecure and confident subgroup (i.e., highest intervention effect in confident instead of insecure subgroup). All analyses were done using R version 3.6.0^51^.

## Results

### Model population

The baseline parameter values in the model were based on data from 810 participants in the iMPaCT study who completed the baseline questionnaire (76% of all participants, Supplementary Material, table S2). Based on the baseline iMPaCT data (Table 1), 10% of the model population started in the low-impulsivity, 38% in the condom-using, 21% in the insecure, and 31% in the confident subgroup. After the incorporation of behaviour change in the model, the steady-state subgroup sizes were comparable to the subgroup proportions in the iMPaCT study at one-year follow-up (i.e., without interventions). The steady-state chlamydia prevalence in the model was 13% in the total population, and 12% in the low-impulsivity, 10% in the condom-using, 12% in the insecure, and 19% in the confident subgroup. This was comparable to the iMPaCT data: the steady-state prevalence in each subgroup in the model fell within the 95% confidence interval of the chlamydia positivity rates found in each subgroup in the data (Table 1, Supplementary Material Figure S4).

### Non-differential versus differential intervention effects

**Figure 1:**
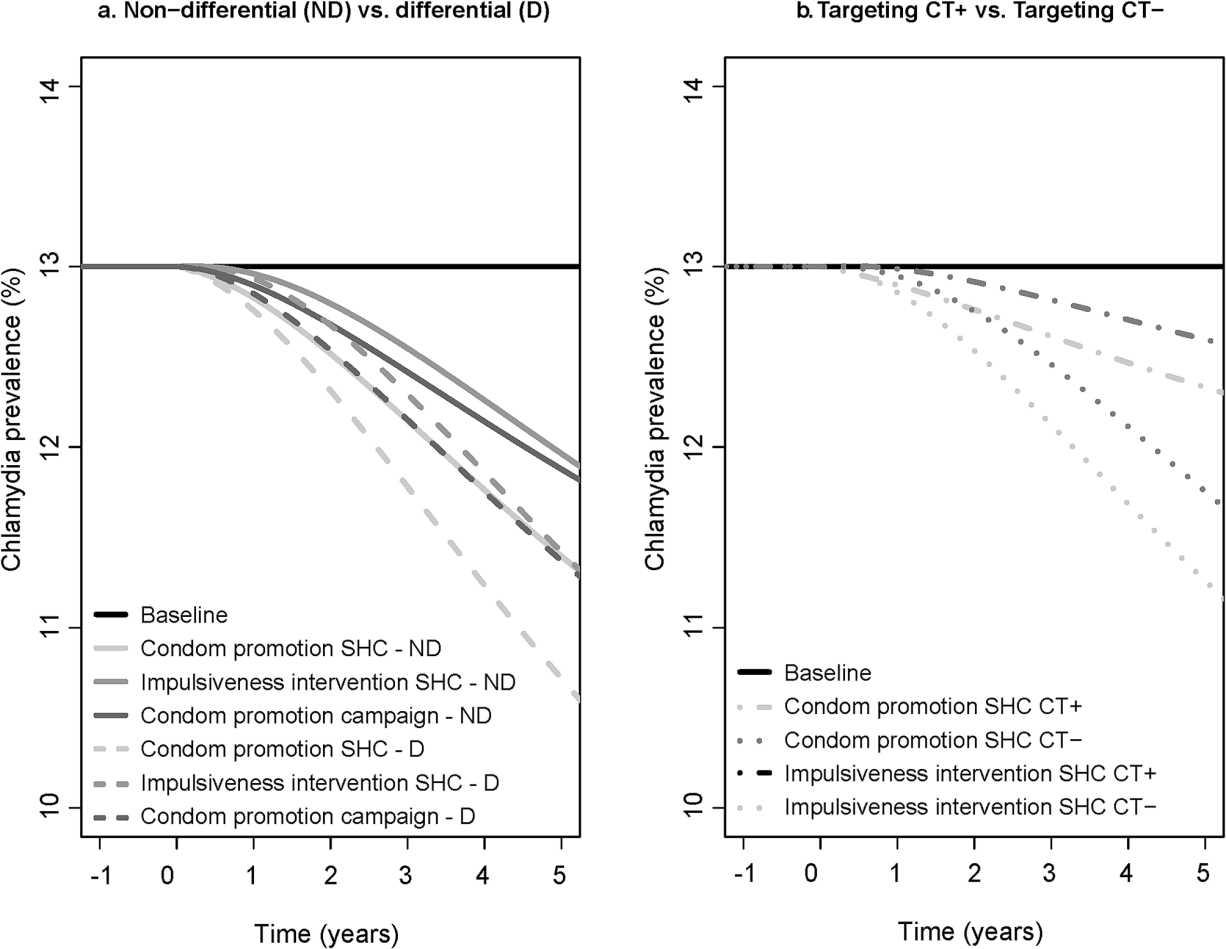
In the left panel **(a)**, the impact of introducing condom promotion at SHC, an impulsiveness intervention at SHC, and condom promotion campaign, assuming a non-differential (ND) intervention effect (solid lines), and a differential (D) intervention effect (dashed lines) on overall chlamydia prevalence for five consecutive years is shown. On the right panel **(b)**, the impact of introducing condom promotion at SHC, and an impulsiveness intervention at SHC with differential intervention effects, assuming that only individuals who were diagnosed with chlamydia were targeted (CT + , dash-dotted lines), and that only individuals who tested chlamydia negative were targeted (CT−, dotted lines), on overall chlamydia prevalence for five consecutive years is shown. Note that the y-axis starts at 10% to better visualize the differences between the interventions.

When assuming non-differential intervention effects, the relative reduction in overall chlamydia prevalence was 12% five years after the introduction of the condom promotion at SHC, 8% after the impulsiveness intervention at SHC, and 9% after the condom promotion campaign. The impact on overall chlamydia prevalence assuming differential intervention effects was higher compared to assuming non-differential intervention effects (Fig. 1A). When assuming differential intervention effects, the relative reduction in overall chlamydia prevalence was 18% five years after the introduction of the condom promotion at SHC, 12% after the impulsiveness intervention at SHC, and 13% after the condom promotion campaign. Irrespective of the assumed intervention effects, the impact of the condom promotion at SHC on overall chlamydia prevalence was larger compared to the impulsiveness intervention at SHC, and the condom promotion campaign. Approximately ten years after the introduction of the interventions, the impact on chlamydia prevalence stabilizes (Supplementary material Figure S5).

**Figure 2:**
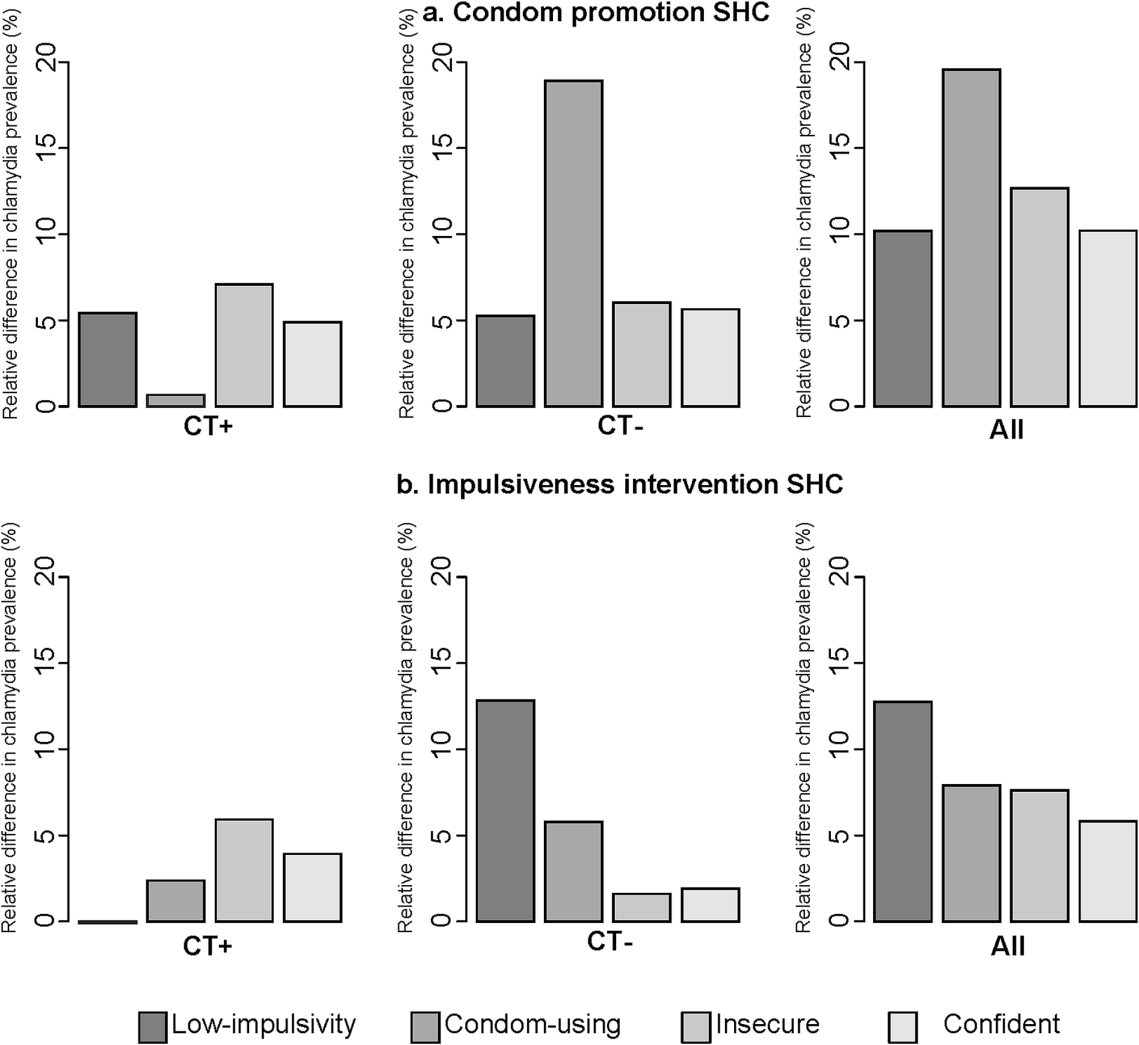
Impact on chlamydia prevalence five years after the introduction of condom promotion at SHC **(a)**, and of the impulsiveness intervention at SHC **(b)** with differential intervention effects, targeted at only at individuals who tested chlamydia positive (CT +), only at individuals who tested chlamydia negative (CT−), and targeted at all tested (All).

The impact of differential intervention effects on subgroup-specific chlamydia prevalence after the condom promotion at SHC, and the condom promotion campaign intervention was largest in the condom-using subgroup (Fig. 2A, right panel ‘All’), due to an increase in size in this group (Supplementary material Figure S6). Similarly, the relative reduction in chlamydia prevalence after the impulsiveness-reducing intervention was largest in the low-impulsivity group in (Fig. 2B, right panel ‘All’). The impact of the condom promotion campaign was larger in the insecure and confident subgroups (relative reduction of 15% in the insecure and 12% in the confident subgroup), compared to the low-impulsivity and condom-using subgroup (relative reduction of 10% in low-impulsivity, and 5% in condom-using subgroup).

### Targeting interventions according to chlamydia test result

The impact of condom promotion at SHC and impulsiveness intervention at SHC with differential intervention effects, targeting only individuals who tested chlamydia negative was larger compared to targeting only individuals who were diagnosed with chlamydia (Fig. 1B). The relative reduction in overall chlamydia prevalence after condom promotion at SHC was 13% targeting only individuals who tested chlamydia negative, and 5% targeting only individuals who were diagnosed with chlamydia. After the impulsiveness intervention at SHC, the relative reduction was 10% targeting only individuals who tested chlamydia negative, and 3% targeting only individuals who were diagnosed with chlamydia in impulsiveness intervention. When the interventions were targeted only at individuals who were diagnosed with chlamydia, the impact was largest in the subgroups with the highest steady-state chlamydia prevalence (i.e., insecure and confident subgroup, Fig. 2A,B). The relative reduction in chlamydia prevalence when the interventions were targeted only at individuals who tested negative was largest in the condom-using and low-impulsivity subgroups.

### Uncertainty analyses

**Figure 3:**
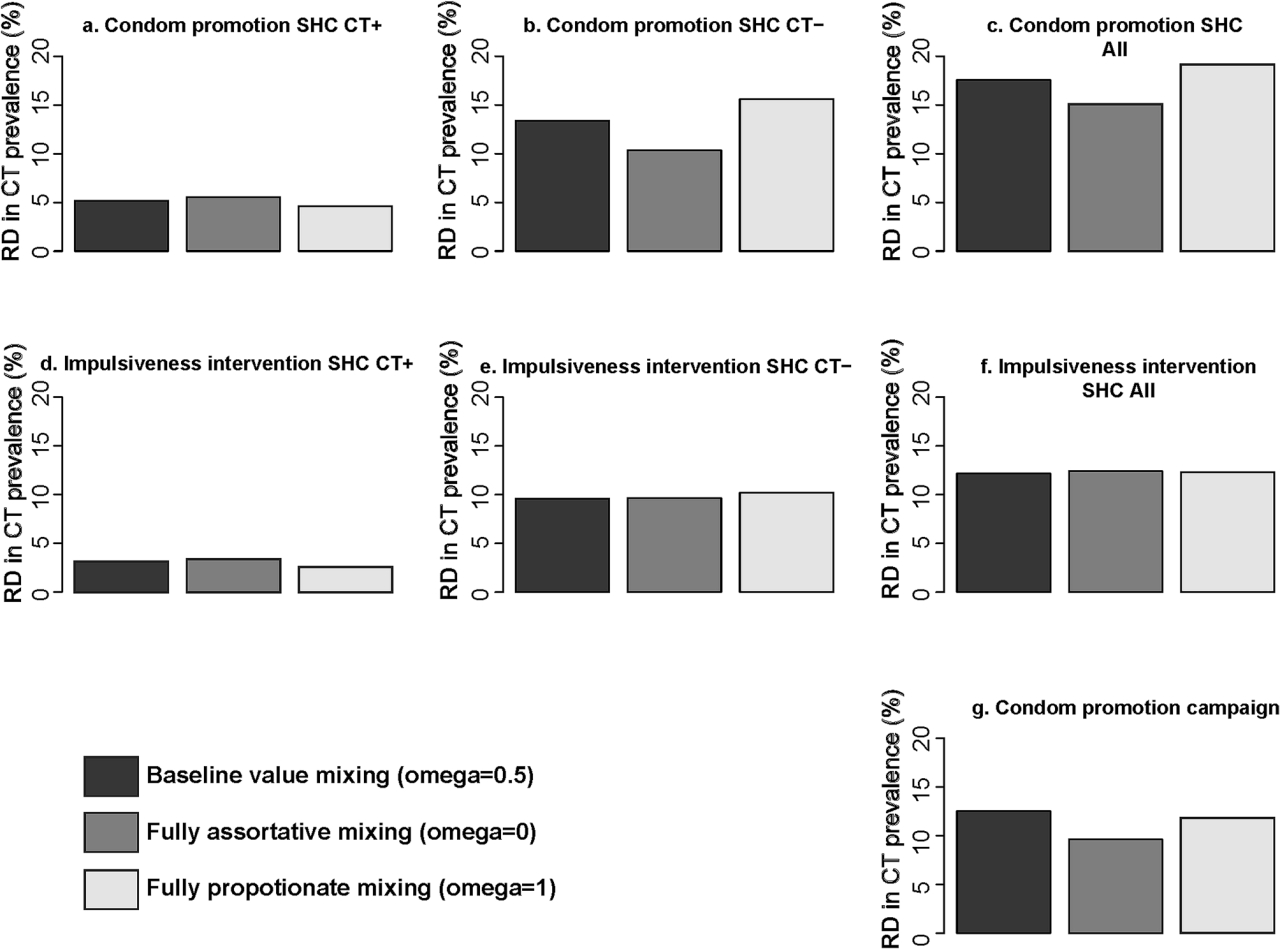
Uncertainty analyses mixing parameter. Impact on overall chlamydia prevalence five years after the introduction of condom promotion at SHC **(a–c)**, impulsiveness intervention at SHC **(d–f)**, targeted at only at individuals who tested chlamydia positive (CT +), only at individuals who tested chlamydia negative (CT−), and targeted at all tested (All), and the condom promotion campaign **(g)**, for different values of the mixing parameter. *CT* *Chlamydia trachomatis*, *CT*+  *Chlamydia trachomatis* positive; *CT*− *Chlamydia trachomatis* negative, *RD* relative difference.

**Figure 4:**
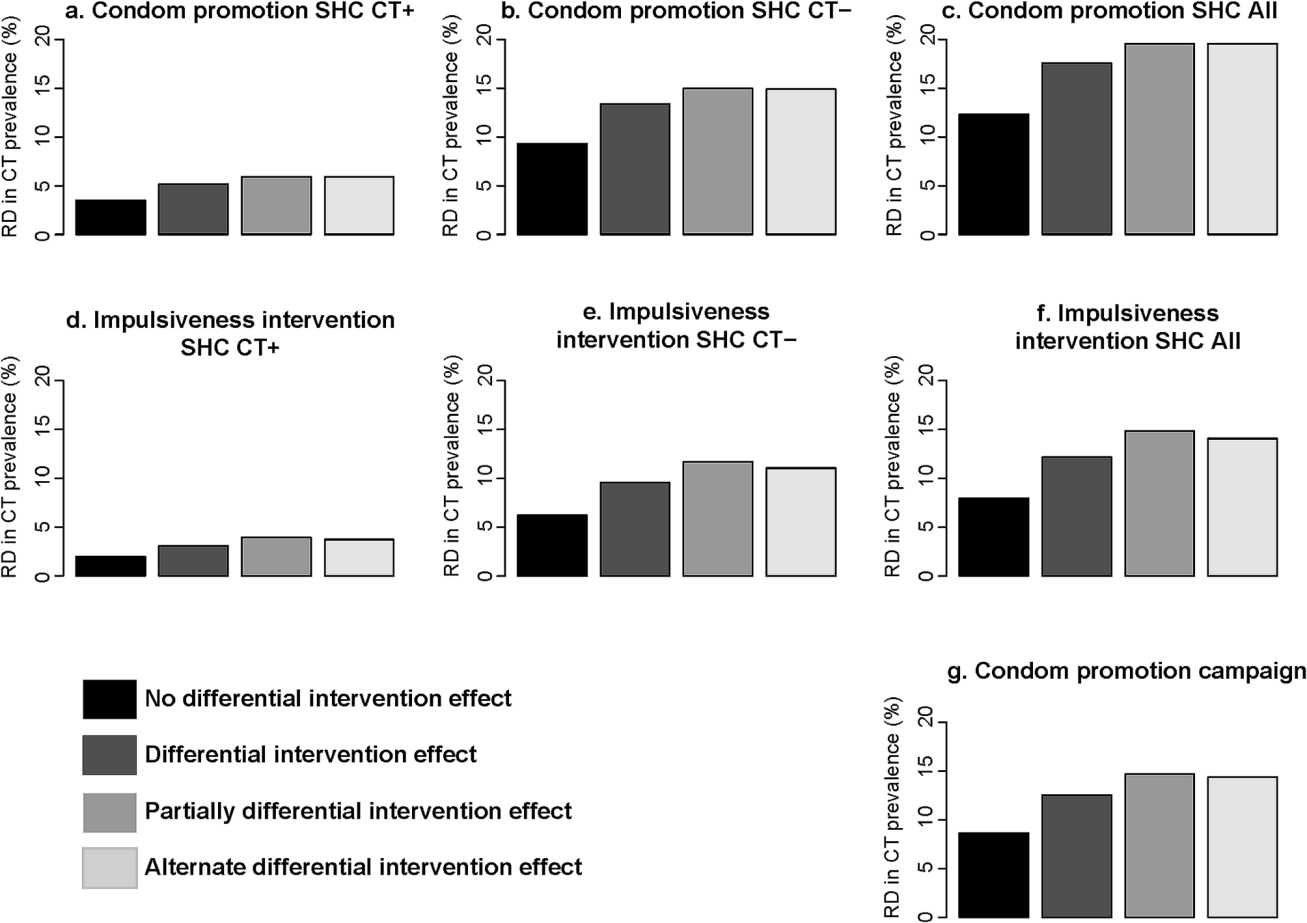
Uncertainty analyses intervention effect. Impact on overall chlamydia prevalence five years after the introduction of condom promotion at SHC **(a–c)**, impulsiveness intervention at SHC **(d–f)**, targeted at only at individuals who tested chlamydia positive (CT+), only at individuals who tested chlamydia negative (CT−), and targeted at all tested (All), and the condom promotion campaign **(g)**, for assuming no differential intervention effect, differential intervention effect, partially differential intervention effect, and alternate differential intervention effect. *CT* *Chlamydia trachomatis*, *CT*+  *Chlamydia trachomatis* positive; *CT*− *Chlamydia trachomatis* negative, *RD* relative difference.

Changing the mixing parameter from assortative to proportionate mixing had hardly any effect on the estimated impact on chlamydia prevalence after the impulsiveness intervention (Fig. 3). However, it did influence the relative reduction in chlamydia prevalence after the condom promotion interventions: assortative mixing decreased the impact on overall chlamydia prevalence, whereas proportionate mixing increased the impact of the interventions. When only individuals who were diagnosed with chlamydia were targeted, assuming assortative mixing slightly increased the impact, whereas proportionate mixing slightly decreased the impact on chlamydia prevalence compared to the main analysis. Changing the assumptions on the differential intervention effect had little effect on the estimated impact on chlamydia prevalence in all intervention scenarios (Fig. 4).

## Discussion

Behavioural interventions tailored to subgroup-specific psychological characteristics based on model assumptions reduced chlamydia prevalence more effectively compared to targeting the same intervention to the total STI clinic population without keeping subgroup-characteristics in mind. Furthermore, a behavioural intervention aimed at increasing self-efficacy, social norms, attitudes and intentions towards condom use, was more effective than an intervention aimed at increasing health goals and decreasing impulsive behaviour in terms of reducing overall chlamydia prevalence in the model. The relative reduction in overall chlamydia prevalence when targeting tailored interventions only to individuals who tested negative was much larger compared to targeting only those who were diagnosed with chlamydia.

A strength of this study is that, to our knowledge, this is the first mathematical modelling study that defines subgroups for STI based on multiple psychological and behavioural characteristics, allowing for multiple target variables for behavioural interventions. Furthermore, the model was informed by real-life longitudinal data on psychological determinants, testing behaviour, and test results. This enabled us to incorporate behaviour change in the model by estimating transition rates based on data.

There were also several limitations. First, no data was available on the actual effect size of the behavioural interventions. However, changing the assumptions on the differential intervention effects in the uncertainty analyses had hardly any effect on the relative reduction in chlamydia prevalence, indicating robust estimation of the impact of the interventions. Furthermore, this may also be a strength of this modelling study, because we estimated the effects of hypothetical interventions (based on reasonable assumptions), which could be used to prioritize interventions to be implemented and evaluated in practice. Second, it was difficult to obtain subgroup-specific chlamydia prevalence in the model that matched the positivity rates observed in the data, which may have led to an overestimation of the intervention impact on chlamydia prevalence. This might be explained by factors for which no data was available, such as psychological and behavioural characteristics of partners of the participants, or dominant behaviour in partnerships (e.g., in a partnership, behaviour of individual from one subgroup might be more dominant than the behaviour of an individual from another subgroup^53^). Third, the low number of male participants hampered stratified analyses for gender. However, although behavioural parameters were not different between males and females in the model, the possible underlying psychological mechanisms that explain gender differences in behaviour are included in the model, as the proportion of males and females are different in each subgroup^50^. Furthermore, there is uncertainty about gender differences in biological parameters for chlamydia transmission, such as the duration of infection and natural clearance, and the transmission probability. Last, we used data from one-year follow-up to inform the transition probabilities in the model that were assumed to be independent of testing, but all the participants had been tested for STI at least once in the past year. Nevertheless, participants in the iMPaCT study were recruited at the SHC, but only visited the SHC at baseline, with the exception of a few participants who reported to have been tested again in the year following baseline. Therefore, we used data at one-year follow-up to model behaviour change of individuals who do not visit the SHC. The results of the current study may have limited generalizability for the population aged ≥ 25 years, but as young heterosexuals aged < 25 years are viewed to be at higher risk of acquiring chlamydia^35,36^, the conclusions are applicable to a clinically relevant subgroup.

Similar to the results of our study, previous modelling efforts in South Africa^54,55^, and the Netherlands^53^ estimated that interventions increasing condom use would result in a significant reduction in chlamydia prevalence. However, it is difficult to compare the estimated reduction in chlamydia prevalence from these modelling studies with our results for several reasons. For example, the model populations were different (general population aged 15–49 years vs. heterosexual SHC visitors aged 18–24 years in our study). As SHC visitors are usually at higher risk than the general population^35,36^, behavioural parameters and chlamydia prevalence were different. Furthermore, we used extensive data to define the subgroups based on psychological and behavioural characteristics, enabling us to estimate the impact of interventions tailored to multiple subgroups, whereas incorporating heterogeneity in sexual behaviour in the other modelling studies was limited to defining a core group with high partner change rates.

The findings that tailored interventions based on model assumptions more effectively reduced overall chlamydia prevalence compared to non-tailored interventions can be explained by changes in the size of subgroups. As characteristics of the subgroups are related to chlamydia transmission, changing the size of subgroups is equal to changes in sexual behaviour and chlamydia risk. The more people who change their behaviour and end up in lower risk subgroups (due to the assumed effects of tailored interventions), the greater the reduction in chlamydia prevalence. The larger impact of condom promotion at SHC on chlamydia prevalence compared to the impulsiveness intervention at SHC may also be explained by the group size, since the baseline group size of the condom-using subgroup was larger compared to the low-impulsivity subgroup.

Since condom promotion at the SHC reduced overall chlamydia prevalence more effectively in the model than a condom promotion campaign, SHC might be a good location to implement behavioural interventions tailored to subgroup-characteristics. Risk-reduction counselling at SHC, such as motivational interviewing after a positive chlamydia test result, is already an integral part of the national guidelines for STI management in many Western countries^26,29,56^. The reason for this is that individuals who were diagnosed with chlamydia are at high risk of acquiring chlamydia again within a year^35,57,58^. Our model results showed that targeting behavioural interventions at all tested high-risk individuals, including both individuals who tested chlamydia positive as well as those who tested negative, should be a part of STI management as well in order to reduce chlamydia prevalence most effectively. However, as sufficient resources to deliver behavioural interventions face-to-face to all tested individuals (e.g., during an STI test consultation) are often not available, online interventions might be a good alternative^34,59,60^. For example, a validated set of questions to assess certain psychological characteristics may be added to the process of making an appointment online (i.e., before testing). This extra information could be used to deliver tailored online interventions at the SHC, such as a video-based intervention during the online intake assessment, or after retrieving the chlamydia test results online^34,59,60^.

We showed that, based on model assumptions, condom promotion at the SHC, targeting self-efficacy, social norms, attitudes and intentions towards condom use, most effectively reduced overall chlamydia prevalence. Future work could focus on evaluating the effectiveness of such interventions. Data collection is needed on the proportion of the SHC population receiving the intervention (online or during test/treatment consultation), and the proportion of behaviour change and chlamydia diagnoses after the intervention. The gold standard for evaluating the effectiveness of interventions would be to conduct randomized controlled trials (RCTs). This would mean that the participants will be assigned to an intervention and control arm randomly. Furthermore, before setting up large-scale, time consuming RCTs, it might be useful to gain insights into how successful implementation of interventions, particularly online interventions, in the SHC setting could be achieved by conducting a pilot study. For example, a pilot study could be performed to find out what specific set of questions is needed to characterize an individuals’ profile with as few questions as possible without creating barriers to complete the online intake assessment for an STI test.

To conclude, behavioural interventions tailored to psychological and behavioural characteristics of an individual may be more successful in achieving risk-reducing behaviour than non-tailored interventions, and consequently, reduce chlamydia prevalence more effectively.

## Supplementary Information

Supplementary Information.

## Data Availability

Data and R codes are available upon request.
