# Supplementary material for: Modelling the impact of tailored behavioural interventions on chlamydia transmission
Source: Sci Rep. 2021 Jan 25;11:2148. doi: 10.1038/s41598-021-81675-w (PMC7835240; doi:10.1038/s41598-021-81675-w)
Supplement: Supplementary file 1 — Supplementary Information. [file 41598_2021_81675_MOESM1_ESM.pdf]

Supplementary Material

Modelling the impact of tailored behavioural interventions on chlamydia transmission

Daphne A. van Wees, Chantal den Daas, Mirjam E.E. Kretzschmar, Janneke C.M. Heijne

Contents

S1. Description of the model 2
S1.1. Schematic overview of the chlamydia infection process 2
S1.2. Behaviour change after testing and independent of testing 4
S1.3. Differential equations 8
S1.4. Behavioural parameters informed by the iMPaCT study 12
S1.4.1. Calculation pair formation and separation rates 12
S1.4.2. Calculation of the number of partners per year and number of one-night stands 13
S1.4.3. One-night stands in single state and in pairs 14
S1.4.4. Calculation number of sex acts 14
S2. Additional methods 15
S2.1. Calculation of transition probabilities in the intervention scenarios 15
S2.2. Calculation of the reduction in chlamydia prevalence 19
S3. Additional results 21
References 24

List of figures

Figure S1. Schematic overview of the chlamydia infection process 3
Figure S2. Possible transitions from one subgroup to another after testing. 5
Figure S3. Example calculation of the relative reduction in chlamydia prevalence 20
Figure S4. Model fit of chlamydia prevalence to iMPaCT data in each subgroup 21
Figure S5. The impact of introducing interventions on chlamydia prevalence long-term 22
Figure S6. Estimated impact of interventions scenarios on the group sizes 23

List of tables

Table S1. Psychological and behavioural characteristics of the subgroups 4
Table S2. Symbols, baseline values and description of all parameters 6
Table S3. Transition probabilities as identified in the latent transition analysis 7
Table S4. Calculations of the intervention transition probabilities. 17
Table S5. Intervention effect in each subgroup for different intervention scenarios 19

S1. Description of the model

S1.1. Schematic overview of the chlamydia infection process

A schematic overview of the chlamydia infection process in a pair compartmental model with a susceptible-infected-susceptible (SIS) structure representing heterosexuals aged 18 to 24 years is provided in Figure S1. In the model, individuals can either be susceptible (S) or infected (I). Furthermore, the subgroups described in text S1.1 and corresponding behavioural parameters were incorporated in the model. The subgroup of an individual in the model is denoted by subscript $i \in \{1,2,3,4\}$ for females and $j \in \{1,2,3,4\}$ for males. The indices i (and j) represent low-impulsivity (i = 1), condom-using (i = 2), insecure (i = 3), and confident subgroup (i = 4) individuals, which could be differentiated from each other by psychological and behavioural characteristics (Table S1). The proportion of males and females in the total population and in all the subgroups was assumed to be equal.

Individuals can either be single (upper and lower two compartments in Figure S1, denoted by X in the model equations) or in a pair (compartments in the middle row in Figure S1, denoted by P in the model equations). Single females and males can form pairs of two susceptible individuals ($S_i\, S_j$), susceptible and infected individuals ($S_i\, I_j$ or $I_i\, S_j$), or two infected individuals ($I_i\, I_j$) with pair formation rate ρ_i (=ρ_j), and pairs can break up with pair separation rate σ_{ij}. The pair formation and separation processes are independent of the infection status. Furthermore, singles and individuals in pairs can have one-time sexual encounters further referred to as 'one-night stands' with rate $\eta_i\, \pi_i^X$ (=$\eta_j\, \pi_j^X$) for singles and rate $\eta_i\, \pi_i^P$ (=$\eta_j\, \pi_j^P$) for pairs, whereη_i is the number of one-night stands per year in each subgroup, π_i^X is the proportion of the number one-night stands per year occurring in a single state, and π_i^P is the proportion of the number one-night stands per year for individuals who are already in a partnership. Individuals can become infected in a pair of a susceptible and infected female in subgroup i and a male in subgroup j with rate $\beta\varepsilon_{ij}\varphi_{ij}$, where β denotes the transmission probability per sex act, ε_{ij} the proportion of sex acts without a condom per year for each subgroup combination, and φ_{ij} the number of sex acts per year for each subgroup combination. Infection from

a one-night stand is described with the term $\beta \varepsilon_{ij} \frac{I_i}{N_i}$, where $\frac{I_i}{N_i}$ (or $\frac{I_j}{N_j}$) is the proportion of infected opposite sex individuals in the total population. All individuals can become susceptible again after natural clearance of infection with rate γ or after testing and treatment with rate $\alpha_i = \alpha_j$. In the model, a 100% test sensitivity, 100% treatment acceptance by everyone who is diagnosed, and a 100% treatment effectiveness, was assumed. Table S2 and text S1.3, S1.4 and S1.5 show descriptions, symbols, and baseline values of the infection and behavioural parameters in each subgroup included in the model and the differential equations.

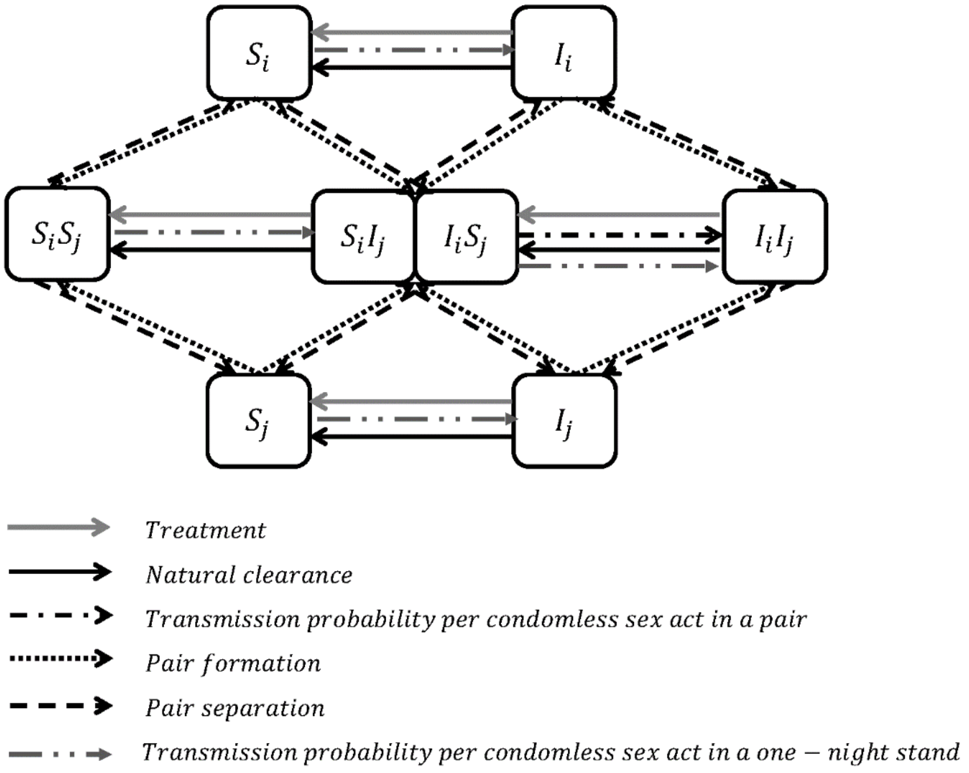

Figure S1. Schematic overview of the chlamydia infection process in a pair compartmental model
Abbreviations: I = Infected; S = Susceptible.

Table S1. Psychological and behavioural characteristics of the subgroups

**Variable**	**Subgroup**			
	Low-impulsivity (%)	*Condom-using (%)*	*Insecure (%)*	*Confident (%)*
High number of partners[a]	13	**61**	**71**	**81**
Consistent condom use[b]	9	**97**	**42**	**27**
High health goals	73	**89**	**30**	**23**
High attitudes[c]	77	81	**54**	**29**
High intentions[d]	56	**78**	**35**	**13**
High self-efficacy	59	**76**	**25**	43
High social context[e]	69	**80**	**55**	**55**
High anticipated stigma	40	**51**	**84**	**24**
High anticipated shame	63	66	**87**	**27**
High impulsiveness	22	**38**	**76**	**59**
High knowledge[f]	62	57	58	65
High self-esteem	76	74	**39**	**82**
High risk perception for chlamydia	19	**31**	**53**	**63**

[a] Reporting ≥3 partners in the past six months
[b] Condom use in general, consistent=usually/always
[c] Attitudes regarding prevention of chlamydia
[d] Intentions regarding condom use and STI testing
[e] Social context regarding condom use and STI testing (i.e.. social support, social and subjective norms)
[f] Knowledge regarding sexual health, prevention of chlamydia and consequences of chlamydia diagnosis
Footnote: Statistically significant differences in univariate logistic regression are shown in bold when p-value < 0.05, low-impulsivity = reference group.
[Adapted from: van Wees et al., 2019[1]]

S1.2. Behaviour change after testing and independent of testing

Individuals can get tested at rate $\alpha_{\hat{\imath}}$ $(= \alpha_{\hat{\jmath}})$ per year and move to another subgroup after testing with transition probability $\lambda_{\hat{\imath}i}$ (=$\lambda_{\hat{\jmath}j}$) for infected individuals and $\delta_{\hat{\imath}i}$ (=$\delta_{\hat{\jmath}j}$) for susceptible individuals (see Figure S2), where α is the testing uptake rate per year, λ is the transition probability for infected individuals, δ is the transition probability for susceptible individuals, $\hat{\imath}$ is the subgroup they were in before testing, and i is the subgroup they moved to after testing. For example, an infected female individual moves from the confident subgroup ($\hat{\imath}$=4) to the condom-using subgroup (i=2) after testing with rate $\alpha_4\lambda_{42}$. Furthermore, individuals can also move to another subgroup if they were not tested, with rate $\kappa_{\hat{\imath}}\tau_{\hat{\imath}i}$ (=$\kappa_{\hat{\jmath}}\tau_{\hat{\jmath}j}$), where κ is the behaviour change rate per year independent of testing, τ is the transition probability independent of testing, $\hat{\imath}$ is the subgroup they were in before changing their behaviour, and i is the subgroup they moved to after changing their behaviour. For instance, a male

individual (susceptible or infected) moves from the insecure subgroup ($\hat{j}$=3) to the low-impulsivity subgroup (j=1) with rate $\kappa_3\tau_{31}$), per year. Also, influx of susceptible singles and efflux was added to the model, to retain relative stability of the subgroup sizes. Thus, individuals are introduced into the model population at age 18, with rate $\mu N_i = \mu N_j$, and are removed from the model population when they turn 25 with rate μ. Table S2 and Table S3 show descriptions, symbols, and baseline values of the intervention parameters in each subgroup included in the model and the differential equations.

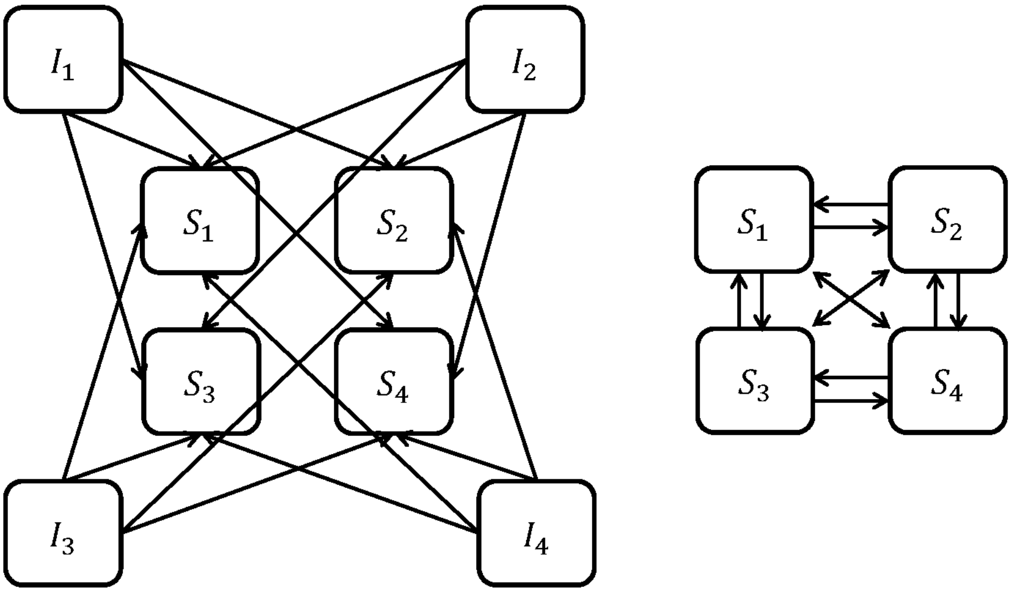

Figure S2. Possible transitions from one subgroup to another after testing for chlamydia positives (left) and negatives (right).
Abbreviations; I = Infected, S = Susceptible; 1=Low-impulsivity subgroup; 2=Condom-using subgroup; 3=Insecure subgroup; 4=Confident subgroup

Table S2. Symbols, baseline values and description of all parameters of the model.

Symbol	Description	Baseline values				Source
Infection parameters						
$1/\gamma$	Duration of untreated infection, years	1				[2,3]
β	Transmission probability per sex act	0.25				Fitted
1/μ	Length of time in the model, based on age range 18-24 years	6 years				IMPaCT data
Behavioural parameters						
ω	Mixing parameter to change from fully assortative ($\omega = 0$) to fully proportionate ($\omega = 1$)	0.5				Value agreed upon by the authors
		**Subgroup-specific baseline values**				
		Low-impulsivity	*Condom-using*	*Insecure*	*Confident*	
$\rho_i = \rho_j$	Pair formation rate, per year	5.16	5.93	6.93	10.22	IMPaCT data[a]
$\eta_i = \eta_j$	Number of one-night stands, per year	0	1	1	2	IMPaCT data[b]
$\pi_i^X = \pi_j^X$	Proportion of one-night stands per year in singles	0.67	0.77	0.76	0.71	IMPaCT data[c]
$\pi_i^P = \pi_j^P$	Proportion of one-night stands per year in pairs	0.33	0.23	0.24	0.29	IMPaCT data[c]
$\sigma_i = \sigma_j$	Pair separation rate, per year	2.43	5.05	4.43	5.75	IMPaCT data[a]
$\varphi_i = \varphi_j$	Number of sex acts, per week	1.25	0.75	1	1	IMPaCT data[d]
$\varepsilon_i = \varepsilon_j$	Proportion of condomless sex acts, per year	0.75	0.25	0.75	0.75	IMPaCT data
$\mu N_i = \mu N_j$	Influx of newly susceptible individuals, per year	0.01	0.24	0.39	0.36	Fitted
Intervention parameters						
$\alpha_i = \alpha_j$	Testing uptake rate, per year	0.9	0.1	0.9	0.7	Fitted
$\kappa_i = \kappa_j$	Behaviour change rate independent of testing, per year	1	1	1	1	IMPaCT data

Footnote. The subgroup of an individual in the model is denoted by subscript $i \in \{1,2,3,4\}$ for females and $j \in \{1,2,3,4\}$ for males.
[a] Detailed description of calculations in Text S1.4.1.
[b] Detailed description of calculations in Text S1.4.2.
[a] Detailed description of calculations in Text S1.4.3.
[b] Detailed description of calculations in Text S1.4.4.

Table S3. Transition probabilities after a chlamydia negative or positive test result, and behaviour change independent of testing in the total population, as identified in the latent transition analysis with the iMPaCT data

Symbol	Description				
$\delta_{\hat{\imath}i}/\delta_{\hat{\jmath}j}$	Transition probabilities CT-				
		**iMPaCT 3-week follow-up**			
	**iMPaCT Baseline**	*Low-impulsivity* $(i = 1)$	*Condom-using* $(i = 2)$	*Insecure* $(i = 3)$	*Confident* $(i = 4)$
	Low-impulsivity $(\hat{\imath} = 1)$	0.986	0.000	0.000	0.014
	Condom-using $(\hat{\imath} = 2)$	0.000	0.996	0.000	0.004
	Insecure $(\hat{\imath} = 3)$	0.007	0.000	0.938	0.055
	Confident $(\hat{\imath} = 4)$	0.000	0.019	0.000	0.981
$\lambda_{\hat{\imath}i}/\lambda_{\hat{\jmath}j}$	Transition probabilities CT+				
		**iMPaCT 3-week follow-up**			
	**iMPaCT Baseline**	*Low-impulsivity* $(i = 1)$	*Condom-using* $(i = 2)$	*Insecure* $(i = 3)$	*Confident* $(i = 4)$
	Low-impulsivity $(\hat{\imath} = 1)$	1.000	0.000	0.000	0.000
	Condom-using $(\hat{\imath} = 2)$	0.000	1.000	0.000	0.000
	Insecure $(\hat{\imath} = 3)$	0.000	0.000	0.950	0.050
	Confident $(\hat{\imath} = 4)$	0.026	0.079	0.000	0.895
$\tau_{\hat{\imath}i}/\tau_{\hat{\jmath}j}$	Transition probabilities independent of testing				
		**iMPaCT 1-year follow-up**			
	**iMPaCT Baseline**	*Low-impulsivity* $(i = 1)$	*Condom-using* $(i = 2)$	*Insecure* $(i = 3)$	*Confident* $(i = 4)$
	Low-impulsivity $(\hat{\imath} = 1)$	0.880	0.120	0.000	0.000
	Condom-using $(\hat{\imath} = 2)$	0.235	0.724	0.016	0.025
	Insecure $(\hat{\imath} = 3)$	0.048	0.012	0.898	0.042
	Confident $(\hat{\imath} = 4)$	0.102	0.055	0.004	0.839

Footnote: the sum of the transition probabilities to move from one specific subgroup at baseline to another subgroup at three-week or one-year follow-up is 1 (horizontal).
Abbreviations: CT- = Chlamydia Trachomatis Negative; CT+ = Chlamydia Trachomatis Positive

S1.3. Differential equations

Gender is denoted by subscript *f* for females and *m* for males, and subscript $i \in \{1,2,3,4\}$ denotes the subgroup for females and $j \in \{1,2,3,4\}$ for males. In total, the model has 80 compartments: 8 for single susceptible $(X_f S_i)$ and infected females $(X_f I_i)$, 8 for single susceptible $(X_m S_i)$ and infected $(X_m I_i)$ males, and 64 types of pairs.

The number of singles is given as:

$$X = X_f + X_m = [\sum_{i=1}^{4} (X_f S_i)] + [\sum_{i=1}^{4} (X_f I_i)] + [\sum_{j=1}^{4} (X_m S_j)] + [\sum_{j=1}^{4} (X_m I_j)]$$

The total population is given as:

$$N = X + 2\,[\sum_{i=1}^{4} \sum_{j=1}^{4} \left(PS_i\,S_j + PS_i\,I_j + PI_i\,S_j + PI_i\,I_j\right)]$$

The SIS model is described by the following differential equations:

Equations for **single females:**

$$\frac{dX_f S_i}{dt} = \frac{1}{2}\mu N_i + (\mu + \sigma_{ij})\sum_{j=1}^{4}\left(PS_i\,S_j + PS_i\,I_j\right) - (\mu + \rho_i)X_f S_i + \gamma X_f I_i + \sum_{\hat{\imath}=1}^{4} \alpha_{\hat{\imath}} \lambda_{\hat{\imath} i}\left(X_f I_{\hat{\imath}}\right)$$

$$+ \sum_{\hat{\imath}=1}^{4} \alpha_{\hat{\imath}} \delta_{\hat{\imath} i}\left(X_f S_{\hat{\imath}}\right) + \sum_{\hat{\imath}=1}^{4} \kappa_{\hat{\imath}} \tau_{\hat{\imath} i}\left(X_f S_{\hat{\imath}}\right) - (\alpha_i + \kappa_i) X_f S_i$$

$$- \eta_i\, \pi_i^X\, (X_f S_i) \sum_{j=1}^{4} m_{ij}^X \beta \varepsilon_{ij} \frac{I_j}{N_j}$$

$$\frac{dX_f I_i}{dt} = (\mu + \sigma_{ij})\sum_{j=1}^{4}\left(PI_i\,S_j + PI_i\,I_j\right) + \sum_{\hat{\imath}=1}^{4} \kappa_{\hat{\imath}} \tau_{\hat{\imath} i}\left(X_f I_{\hat{\imath}}\right) - (\mu + \gamma + \rho_i + \alpha_i + \kappa_i) X_f I_i$$

$$+ \eta_i\, \pi_i^X\, (X_f S_i) \sum_{j=1}^{4} m_{ij}^X \beta \varepsilon_{ij} \frac{I_j}{N_j}$$

Equations for **single males:**

$$\frac{dX_mS_j}{dt} = \frac{1}{2}\mu N_j + (\mu + \sigma_{ij}) \sum_{i=1}^{4} \left(PS_i\, S_j + PI_i\, S_j\right) - (\mu + \rho_j)X_mS_j + \gamma X_m I_j$$

$$+ \sum_{\hat{j}=1}^{4} \alpha_{\hat{j}} \lambda_{\hat{j}j} \left(X_m I_{\hat{j}}\right) + \sum_{\hat{j}=1}^{4} \alpha_{\hat{j}} \delta_{\hat{j}j} \left(X_m S_{\hat{j}}\right) + \sum_{\hat{j}=1}^{4} \kappa_{\hat{j}} \tau_{\hat{j}j} \left(X_m S_{\hat{j}}\right) - (\alpha_j$$

$$+ \kappa_j)X_mS_j - \eta_j\, \pi_j^X\, (X_mS_j) \sum_{i=1}^{4} m_{ij}^X \beta \varepsilon_{ij} \frac{I_i}{N_i}$$

$$\frac{dX_mI_j}{dt} = (\mu + \sigma_{ij}) \sum_{i=1}^{4} \left(PS_i\, I_j + PI_i\, I_j\right) + \sum_{\hat{j}=1}^{4} \kappa_{\hat{j}} \tau_{\hat{j}j} \left(X_m I_{\hat{j}}\right) - (\mu + \gamma + \rho_j + \alpha_j + \kappa_j)X_mI_j$$

$$+ \eta_j\, \pi_j^X\, (X_mS_j) \sum_{i=1}^{4} m_{ij}^X \beta \varepsilon_{ij} \frac{I_i}{N_i}$$

Equations for **pairs where the female is susceptible and the male is susceptible (SS):**

$$\frac{dPS_i\, S_j}{dt} = 2\rho_i\, m_{ij} \left(\frac{XfS_i\, XmS_j}{X_j}\right) - (2\mu + \sigma_{ij})PS_i\, S_j + \gamma(PI_iS_j + PS_iI_j)$$

$$+ \sum_{\hat{i}=1}^{4} \alpha_{\hat{i}} \lambda_{\hat{i}i}\, (PI_{\hat{i}}S_j) + \sum_{\hat{j}=1}^{4} \alpha_{\hat{j}} \lambda_{\hat{j}j}\, (PS_iI_{\hat{j}})$$

$$+ \sum_{\hat{i}=1}^{4} \alpha_{\hat{i}} \delta_{\hat{i}i}\, (PS_{\hat{i}}S_j) + \sum_{\hat{j}=1}^{4} \alpha_{\hat{j}} \delta_{\hat{j}j}\, (PS_iS_{\hat{j}})$$

$$+ \sum_{\hat{i}=1}^{4} \kappa_{\hat{i}} \tau_{\hat{i}i}\, (PS_{\hat{i}}S_j) + \sum_{\hat{j}=1}^{4} \kappa_{\hat{j}} \tau_{\hat{j}j}\, (PS_iS_{\hat{j}}) - \left(a_i + a_j + \kappa_i + \kappa_j\right)PS_iS_j$$

$$- \eta_i\, \pi_i^P\, (PS_i\, S_j) \sum_{j=1}^{4} m_{ij}^P \beta \varepsilon_{ij} \frac{I_j}{N_j} - \eta_j\, \pi_j^P\, (PS_i\, S_j) \sum_{i=1}^{4} m_{ij}^P \beta \varepsilon_{ij} \frac{I_i}{N_i}$$

Equations for **pairs where the female is susceptible and the male is infected (SI):**

$$\frac{dPS_iI_j}{dt} = 2\big(1-\beta\varepsilon_{ij}\big)\rho_i\, m_{ij}\left(\frac{XfS_i\, XmI_j}{X_j}\right) - (2\mu + \sigma_{ij} + \beta\varepsilon_{ij}\varphi_{ij})PS_iI_j + \sum_{\hat{\imath}=1}^{4} \alpha_{\hat{\imath}}\lambda_{\hat{\imath}i}\,(PI_{\hat{\imath}}I_j)$$

$$+ \sum_{\hat{\imath}=1}^{4} \alpha_{\hat{\imath}}\delta_{\hat{\imath}i}\,(PS_{\hat{\imath}}I_j) + \sum_{\hat{\imath}=1}^{4} \kappa_{\hat{\imath}}\tau_{\hat{\imath}i}\,(PS_{\hat{\imath}}I_j) + \sum_{\hat{\jmath}=1}^{4} \kappa_{\hat{\jmath}}\tau_{\hat{\jmath}j}\,(PS_iI_{\hat{\jmath}})$$

$$-\big(\gamma + a_i + a_j + \kappa_i + \kappa_j\big)PS_iI_j + \eta_j\,\pi_j^P\,(PS_i\,S_j)\sum_{i=1}^{4} m_{ij}^P\beta\varepsilon_{ij}\frac{I_i}{N_i}$$

$$-\ \eta_i\,\pi_i^P\,(PS_i\,I_j)\sum_{j=1}^{4} m_{ij}^P\beta\varepsilon_{ij}\frac{I_j}{N_j}$$

Equations for **pairs where the female is infected and the male is susceptible (IS):**

$$\frac{dPI_i\,S_j}{dt} = 2\big(1-\beta\varepsilon_{ij}\big)\rho_i\, m_{ij}\left(\frac{XfI_i\, XmS_j}{X_j}\right) - (2\mu + \sigma_{ij} + \beta\varepsilon_{ij}\varphi_{ij})PI_iS_j$$

$$+ \sum_{\hat{\jmath}=1}^{4} \alpha_{\hat{\jmath}}\lambda_{\hat{\jmath}j}\,(PI_iI_{\hat{\jmath}}) + \sum_{\hat{\jmath}=1}^{4} \alpha_{\hat{\jmath}}\delta_{\hat{\jmath}j}\,(PI_iS_{\hat{\jmath}})$$

$$+ \sum_{\hat{\imath}=1}^{4} \kappa_{\hat{\imath}}\tau_{\hat{\imath}i}\,(PI_{\hat{\imath}}S_j) + \sum_{\hat{\jmath}=1}^{4} \kappa_{\hat{\jmath}}\tau_{\hat{\jmath}j}\,(PI_iS_{\hat{\jmath}}) - \big(\gamma + a_i + a_j + \kappa_i + \kappa_j\big)PI_iS_j$$

$$+ \eta_i\,\pi_i^P\,(PS_i\,S_j)\sum_{j=1}^{4} m_{ij}^P\beta\varepsilon_{ij}\frac{I_j}{N_j} - \eta_j\,\pi_j^P\,(PI_i\,S_j)\sum_{i=1}^{4} m_{ij}^P\beta\varepsilon_{ij}\frac{I_i}{N_i}$$

Equations for **pairs where the female is infected and the male is infected (II):**

$$\frac{dPI_i\,I_j}{dt} = 2\rho_i\, m_{ij}\left(\frac{XfI_i\,XmI_j}{X_j}\right) + \; 2\beta\varepsilon_{ij}\rho_i\, m_{ij}\left(\frac{XfS_i\,XmI_j}{X_j}\right) + \; 2\beta\varepsilon_{ij}\rho_i\, m_{ij}\left(\frac{XfI_i\,XmS_j}{X_j}\right)$$

$$+ \left(\beta\varepsilon_{ij}\varphi_{ij}\right)\left(PS_iI_j \; + PI_iS_j\right) + \sum_{\hat{\imath}=1}^{4} \kappa_{\hat{\imath}}\tau_{\hat{\imath}i}\,(PI_{\hat{\imath}}I_j) + \sum_{\hat{\jmath}=1}^{4} \kappa_{\hat{\jmath}}\tau_{\hat{\jmath}j}\,(PI_iI_{\hat{\jmath}})$$

$$-\,(2\mu + \sigma_{ij} + \gamma + a_i + \; a_j + \; \kappa_i + \kappa_j)PI_iI_j \; + \; \eta_i\,\pi_i^P\,(PS_i\,I_j)\sum_{j=1}^{4} m_{ij}^P\beta\varepsilon_{ij}\frac{I_j}{N_j}$$

$$+ \;\; \eta_j\,\pi_j^P\,(PI_i\,S_j)\sum_{i=1}^{4} m_{ij}^P\beta\varepsilon_{ij}\frac{I_i}{N_i}$$

The 4 x 4 mixing matrix M = $(m_{ij})_{i,j\,\in\{1,2,3,4\}}$ for partnerships >1 day, and M$^{\eta}$ = $(m_{ij}^{X})_{i,j\,\in\{1,2,3,4\}}$ for one-night stands was defined as follows

$$m_{ij} = \;\omega\left(\frac{\rho_j\,X_j}{\rho_1X_1 + \,\rho_2X_2 \,+ \,\rho_3X_3 + \,\rho_4X_4}\right) + (1 - \;\omega)\theta_{ij} \qquad \text{with } \theta_{ij} = \begin{cases} 1\,for\;i = j \\ 0\,for\;i\;\neq j \end{cases}$$

$$m_{ij}^{\eta} = \;\omega\left(\frac{\eta_j\,N_j}{\eta_1N_1 + \,\eta_2\,N_2 + \,\eta_3\,N_3 + \,\eta_4\,N_4}\right) + (1 - \;\omega)\theta_{ij} \qquad \text{with } \theta_{ij} = \begin{cases} 1\,for\;i = j \\ 0\,for\;i\;\neq j \end{cases}$$

where ω is the mixing parameter used to change from fully assortative (ω = 0) to fully proportionate mixing (ω = 1).

S1.4. Behavioural parameters informed by the iMPaCT study

Behavioural parameters, including the number of partners per year, the number of one-night stands per year, the proportion of one-night stands that occurred in a single state and in pairs, duration of partnerships, and the fraction of the population in a partnership, were informed by the iMPaCT data. As the model assumed serial monogamy (i.e., no overlap of partnerships/concurrency, except for one-night stands that occurred in pairs), data from participants who reported that the second most recent partnership overlapped with the most recent partnership (i.e., ≥1 day overlap, n=183) were excluded from all the calculations described in sections 1.4.1, 1.4.2 and 1.4.3.

S1.4.1. Calculation pair formation and separation rates

The number of partners per year, and the fraction of the population in a partnership in the low-impulsivity, insecure, condom-using, and confident subgroup in the iMPaCT data were used to derive the pair formation rate and the pair separation rate. In the model, the total population size of each subgroup is given by $N_i = X_i + 2P_i$, where the subgroup of an individual (male or female) in the model is denoted by subscript $i \in \{1,2,3,4\}$. The fraction of the population in a partnerships, and in a single state, the number of (new) partners per year, and the pair separation and pair formation rates were calculated for each subgroup separately:

$2P_i = 2\,\frac{\rho_i}{2(\rho_i + \sigma_i)}$ = Fraction of subgroup in a partnership based on iMPaCT data

$X_i = \frac{\sigma_i}{(\rho_i + \sigma_i)}$ = Fraction of subgroup single based on iMPaCT data

$X_i\,\rho_i$ = Number of new partners per year in each subgroup

$X_i\,\rho_i + P_i =$ Number of partners per year in each subgroup based on iMPaCT data

σ_i = pair separation rate

ρ_i = pair formation rate

This corresponds to an average partnership duration in each subgroup of $1/\sigma_i$ (Table 1 and Table S2). The parameter values for average partnership duration in each subgroup in the model were similar to the median partnership duration in each subgroup in the iMPaCT data (see main text, table 1). The

median partnership durations in table 1 were reported, excluding participants who reported that their most recent partner was a one-night stand (n=239) as these are incorporated differently (see section 1.4.2 and 1.4.3).

In partnerships of individuals from different groups, the mean of σ_i and σ_j were used, resulting in 16 different pair separation rate combinations. For example, when a female from the low-impulsivity subgroup forms a partnership with a male from the confident subgroup, the corresponding value for the pair separation rate (σ_{14}) was calculated as follows

$$\sigma_{14} = \frac{\sigma_1 + \sigma_4}{2}$$

S1.4.2. Calculation of the number of partners per year and number of one-night stands

The number of one-night stands was calculated using the iMPaCT data. If the reported partnership duration was one day, and the participant reported that the partnership had ended, it was defined as a one-night stand. As the duration of partnerships was only reported for the two most recent partners, the number of one-night stands per year was calculated by multiplying the total number of reported partners in the past year by the proportion of the two most recent partnerships being a one-night stand. If the two most recent partners were not a one-night stand (duration partnership >1 day), the reported number of partners was multiplied by 0 (i.e., no one-night stands). In this case, none of the reported partners were assumed to be no one-night stands. If one of the two most recent partners was a one-night stand (duration partnership ≤1 day), the reported number of partners was multiplied by 0.5. For example, if a participant reported to have had 4 partners in the past year, 2 of these partners were assumed to be one-night stands, and the other 2 partners were assumed to be no one-night stands. If the two most recent partners were both a one-night stand (duration partnership ≤1 day), the reported number of partners was multiplied by 1. For example, if a participant reported to have had 4 partners in the past year, all 4 partners were assumed to be one-night stands, and zero partners were no one-night stands. Subsequently, the median number of one-night stands and median number of partners per year (i.e., excluding partners that were reported to be one-night stands) were calculated for each subgroup.

S1.4.3. Proportion of one-night stands in single state and in pairs

In the previous paragraph (text S1.4.3) we explained how the number of partners per year (excluding one-night stands) and the number of one-night stands per year were calculated. In this paragraph, we elaborate on the proportion of those one-night stands occurring in a single state and in a pair. As this is a deterministic model, and not agent-based, we were not able to incorporate disproportionately high proportions of one-night stands while in a single state. However, we did assume that the proportion of singles having a one-night stand was different from the proportion of individuals in a pair having a one-night stand. A one-night stand in a pair was defined as participants who reported to have had a one-night stand with another partner while they were in a partnership with their most recent partner (i.e., concurrency). One-night stands for singles was defined as participants who reported that their (second) most recent partnership was a one-night stand without overlap with other partnerships. Subsequently, we calculated the proportion of one-night stands that occurred in a single state or in pairs in each subgroup. For example, if an individual in the low impulsivity subgroup had 3 one-night stands, 1 of those one-night stands occurred in a pair (i.e., 33%, see table S2), and the other two in a single state (67%).

S1.4.4. Calculation number of sex acts

The number of sex acts in the past four weeks as reported in the iMPaCT data, was used to calculate the number of sex acts per week for each subgroup in the model. The reported number of sex acts in the past four weeks was adjusted for the number of weeks they had sex in the past four weeks (based on the most recent sex act). If most recent sex act was:

- <1 week ago: number of sex acts per week = $\frac{\text{number of reported sex acts in the past 4 weeks}}{4}$
- 1 week ago: number of sex acts per week = $\frac{\text{number of reported sex acts in the past 4 weeks}}{3}$
- 2 weeks ago: number of sex acts per week = $\frac{\text{number of reported sex acts in the past 4 weeks}}{2}$
- 3 weeks ago: $number\ of\ sex\ acts\ per\ week = \frac{\text{number of reported sex acts in the past 4 weeks}}{1}$
- ≥ 4 weeks ago: $number\ of\ sex\ acts\ per\ week = 0$

S2. Additional methods

S2.1. Calculation of transition probabilities in the intervention scenarios

In the model, the impact of three behavioural intervention scenarios were explored: condom promotion at SHC, an impulsiveness intervention at SHC, and a condom promotion campaign. The condom promotion at SHC and in the condom promotion campaign in the model were aimed at increasing self-efficacy, social norms, attitudes and intentions towards condom use. The impulsiveness intervention was aimed at increasing health goals and decreasing impulsive behaviour.

In the model, the effect of the interventions was assumed to be the same in each subgroup in the intervention scenarios that were not tailored to subgroup-specific characteristics (i.e., non-differential intervention effect). In the tailored intervention scenarios (i.e., differential intervention effect), the effect was assumed to be different in each subgroup, and the size of the effect was based on subgroup-specific characteristics. In the impulsiveness intervention at SHC, we assumed that the proportion of individuals from the other subgroups moving to the low-impulsivity subgroup (i.e., the intervention subgroup) increased. Since impulsive behaviour was highest in the insecure subgroup, and health goals were lowest in the confident subgroup, the proportion of these subgroups moving to the low-impulsivity subgroup was assumed to be larger than in the condom-using subgroup. Similarly, in the condom promotion at SHC and condom promotion campaign, the proportion of individuals from the other subgroups moving to the condom-using subgroup (i.e., the intervention subgroup) increased. As the levels of self-efficacy towards condom use, and social norms, were lowest in the insecure subgroup, and attitudes and intentions were lowest in the confident subgroup, the proportion of these subgroups moving to the condom-using subgroup was assumed to be larger than in the low-impulsivity subgroup. Furthermore, the testing process itself might have a risk-reducing impact on behavioural and psychological characteristics[4,5]. Therefore, we assumed that the intervention effect of the condom promotion campaign, which was not dependent on testing, was lower than the effect of the condom promotion and impulsiveness intervention at SHC.

The intervention effect ($\nu_{\hat{\imath}}$) was defined as the relative proportional change in the original transition probabilities, which resulted in decreased transition probabilities to move to subgroups that are not the intervention subgroup, and in increased transition probabilities to move to the intervention subgroup for each subgroup (table S4 and table S5). The intervention transition probabilities after

testing for individuals who tested chlamydia negative ($\delta_{\hat{\imath}i} = \delta_{\hat{\jmath}j}$), and in individuals who were diagnosed with chlamydia ($\lambda_{\hat{\imath}i} = \lambda_{\hat{\jmath}j}$), and the intervention transition probabilities for behaviour change independent of testing ($\tau_{\hat{\imath}i} = \tau_{\hat{\jmath}j}$) were calculated using the original transition probabilities (table S4). For example, the original transition probability to move from the insecure to confident subgroup after a negative test result ($\delta_{\hat{3}4}$) was 0.055 (table S3). The intervention effect in the insecure subgroup in the condom promotion at SHC with differential intervention effects was assumed to be 0.2 (table S5). The intervention transition probability to move from the insecure to confident subgroup ($\tilde{\delta}_{\hat{3}4}$) was calculated as follows:

$$\frac{\tilde{\delta}_{\hat{3}4}-\delta_{\hat{3}4}}{\delta_{\hat{3}4}} = -\nu_{\hat{3}} = \frac{\tilde{\delta}_{\hat{3}4}-0.055}{0.055} = -0.2,\ \tilde{\delta}_{\hat{3}4} = 0.044$$

When modelling the intervention scenarios, the original transition probabilities in the differential equations ($\delta_{\hat{\imath}i}, \lambda_{\hat{\imath}i}, \tau_{\hat{\imath}i}$) were replaced by the intervention transition probabilities ($\tilde{\delta}_{\hat{\imath}i}, \tilde{\lambda}_{\hat{\imath}i}, \tilde{\tau}_{\hat{\imath}i}$) (see section 1.3).

Table S4. Calculations of the intervention transition probabilities. In the left column, represent the initial subgroups (i.e., the subgroup an individual starts in). The right columns on top, represent the subgroups individuals can move to over time, with a certain transition probability.

**Intervention transition probabilities - Condom promotion at SHC** $(\tilde{\delta}_{\hat{\imath}j})$				
	Low-impulsivity[b] $(i = 1)$	*Condom-using*[b] $(i = 2)$	*Insecure*[b] $(i = 3)$	*Confident*[b] $(i = 4)$
Low-impulsivity[a] $(\hat{\imath} = 1)$	$\frac{\tilde{\delta}_{\hat{1}1} - \delta_{\hat{1}1}}{\delta_{\hat{1}1}} = -\nu_{\hat{1}}$	$1 - (\tilde{\delta}_{\hat{1}1} + \tilde{\delta}_{\hat{1}3} + \tilde{\delta}_{\hat{1}4})$	$\frac{\tilde{\delta}_{\hat{1}3} - \delta_{\hat{1}3}}{\delta_{\hat{1}3}} = -\nu_{\hat{1}}$	$\frac{\tilde{\delta}_{\hat{1}4} - \delta_{\hat{1}4}}{\delta_{\hat{1}4}} = -\nu_{\hat{1}}$
Condom-using[a] $(\hat{\imath} = 2)$	$\frac{\tilde{\delta}_{\hat{2}1} - \delta_{\hat{2}1}}{\delta_{\hat{2}1}} = -\nu_{\hat{2}}$	$1 - (\tilde{\delta}_{\hat{2}1} + \tilde{\delta}_{\hat{2}3} + \tilde{\delta}_{\hat{2}4})$	$\frac{\tilde{\delta}_{\hat{2}3} - \delta_{\hat{2}3}}{\delta_{\hat{2}3}} = -\nu_{\hat{2}}$	$\frac{\tilde{\delta}_{\hat{2}4} - \delta_{\hat{2}4}}{\delta_{\hat{2}4}} = -\nu_{\hat{2}}$
Insecure[a] $(\hat{\imath} = 3)$	$\frac{\tilde{\delta}_{\hat{3}1} - \delta_{\hat{3}1}}{\delta_{\hat{3}1}} = -\nu_{\hat{3}}$	$1 - (\tilde{\delta}_{\hat{3}1} + \tilde{\delta}_{\hat{3}3} + \tilde{\delta}_{\hat{3}4})$	$\frac{\tilde{\delta}_{\hat{3}3} - \delta_{\hat{3}3}}{\delta_{\hat{3}3}} = -\nu_{\hat{3}}$	$\frac{\tilde{\delta}_{\hat{3}4} - \delta_{\hat{3}4}}{\delta_{\hat{3}4}} = -\nu_{\hat{3}}$
Confident[a] $(\hat{\imath} = 4)$	$\frac{\tilde{\delta}_{\hat{4}1} - \delta_{\hat{4}1}}{\delta_{\hat{4}1}} = -\nu_{\hat{4}}$	$1 - (\tilde{\delta}_{\hat{4}1} + \tilde{\delta}_{\hat{4}3} + \tilde{\delta}_{\hat{4}4})$	$\frac{\tilde{\delta}_{\hat{4}3} - \delta_{\hat{4}3}}{\delta_{\hat{4}3}} = -\nu_{\hat{4}}$	$\frac{\tilde{\delta}_{\hat{4}4} - \delta_{\hat{4}4}}{\delta_{\hat{4}4}} = -\nu_{\hat{4}}$
**Intervention transition probabilities – Impulsiveness intervention** $(\tilde{\delta}_{\hat{\imath}j})$				
	Low-impulsivity $(i = 1)$	*Condom-using* $(i = 2)$	*Insecure* $(i = 3)$	*Confident* $(i = 4)$
Low-impulsivity[a] $(\hat{\imath} = 1)$	$1 - (\tilde{\delta}_{\hat{1}2} + \tilde{\delta}_{\hat{1}3} + \tilde{\delta}_{\hat{1}4})$	$\frac{\tilde{\delta}_{\hat{1}2} - \delta_{\hat{1}2}}{\delta_{\hat{1}2}} = -\nu_{\hat{1}}$	$\frac{\tilde{\delta}_{\hat{1}3} - \delta_{\hat{1}3}}{\delta_{\hat{1}3}} = -\nu_{\hat{1}}$	$\frac{\tilde{\delta}_{\hat{1}4} - \delta_{\hat{1}4}}{\delta_{\hat{1}4}} = -\nu_{\hat{1}}$
Condom-using[a] $(\hat{\imath} = 2)$	$1 - (\tilde{\delta}_{\hat{2}2} + \tilde{\delta}_{\hat{2}3} + \tilde{\delta}_{\hat{2}4})$	$\frac{\tilde{\delta}_{\hat{2}2} - \delta_{\hat{2}2}}{\delta S_{\hat{2}2}} = -\nu_{\hat{2}}$	$\frac{\tilde{\delta}_{\hat{2}3} - \delta_{\hat{2}3}}{\delta_{\hat{2}3}} = -\nu_{\hat{2}}$	$\frac{\tilde{\delta}_{\hat{2}4} - \delta_{\hat{2}4}}{\delta_{\hat{2}4}} = -\nu_{\hat{2}}$
Insecure[a] $(\hat{\imath} = 3)$	$1 - (\tilde{\delta}_{\hat{3}2} + \tilde{\delta}_{\hat{3}3} + \tilde{\delta}_{\hat{3}4})$	$\frac{\tilde{\delta}_{\hat{3}2} - \delta_{\hat{3}2}}{\delta_{\hat{3}2}} = -\nu_{\hat{3}}$	$\frac{\tilde{\delta}_{\hat{3}3} - \delta_{\hat{3}3}}{\delta_{\hat{3}3}} = -\nu_{\hat{3}}$	$\frac{\tilde{\delta}_{\hat{3}4} - \delta_{\hat{3}4}}{\delta_{\hat{3}4}} = -\nu_{\hat{3}}$
Confident[a] $(\hat{\imath} = 4)$	$1 - (\tilde{\delta}_{\hat{4}2} + \tilde{\delta}_{\hat{4}3} + \tilde{\delta}_{\hat{4}4})$	$\frac{\tilde{\delta}_{\hat{4}2} - \delta_{\hat{4}2}}{\delta_{\hat{4}2}} = -\nu_{\hat{4}}$	$\frac{\tilde{\delta}_{\hat{4}3} - \delta_{\hat{4}3}}{\delta_{\hat{4}3}} = -\nu_{\hat{4}}$	$\frac{\tilde{\delta}_{\hat{4}4} - \delta_{\hat{4}4}}{\delta_{\hat{4}4}} = -\nu_{\hat{4}}$
**Intervention transition probabilities - Condom promotion campaign** $(\tilde{\tau}_{\hat{\imath}i})$				
	Low-impulsivity $(i = 1)$	*Condom-using* $(i = 2)$	*Insecure* $(i = 3)$	*Confident* $(i = 4)$
Low-impulsivity[a] $(\hat{\imath} = 1)$	$\frac{\tilde{\tau}_{\hat{1}1} - \tau_{\hat{1}1}}{\tau_{\hat{1}1}} = -\nu_{\hat{1}}$	$1 - (\tilde{\tau}_{\hat{1}1} + \tilde{\tau}_{\hat{1}3} + \tilde{\tau}_{\hat{1}4})$	$\frac{\tilde{\tau}_{\hat{1}3} - \tau_{\hat{1}3}}{\tau_{\hat{1}3}} = -\nu_{\hat{1}}$	$\frac{\tilde{\tau}_{\hat{1}4} - \tau_{\hat{1}4}}{\tau_{\hat{1}4}} = -\nu_{\hat{1}}$

Condom-using[a] $(\hat{\imath} = 2)$	$\frac{\tilde{\tau}_{\hat{2}1} - \tau_{\hat{2}1}}{\tau_{\hat{2}1}} = -\nu_{\hat{2}}$	$1 - (\tilde{\tau}_{\hat{2}1} + \tilde{\tau}_{\hat{2}3} + \tilde{\tau}_{\hat{2}4})$	$\frac{\tilde{\tau}_{\hat{2}3} - \tau_{\hat{2}3}}{\tau_{\hat{2}3}} = -\nu_{\hat{2}}$	$\frac{\tilde{\tau}_{\hat{2}4} - \tau_{\hat{2}4}}{\tau_{\hat{2}4}} = -\nu_{\hat{2}}$
Insecure[a] $(\hat{\imath} = 3)$	$\frac{\tilde{\tau}_{\hat{3}1} - \tau_{\hat{3}1}}{\tau_{\hat{3}1}} = -\nu_{\hat{3}}$	$1 - (\tilde{\tau}_{\hat{3}1} + \tilde{\tau}_{\hat{3}3} + \tilde{\tau}_{\hat{3}4})$	$\frac{\tilde{\tau}_{\hat{3}3} - \tau_{\hat{3}3}}{\tau_{\hat{3}3}} = -\nu_{\hat{3}}$	$\frac{\tilde{\tau}_{\hat{3}4} - \tau_{\hat{3}4}}{\tau_{\hat{3}4}} = -\nu_{\hat{3}}$
Confident[a] $(\hat{\imath} = 4)$	$\frac{\tilde{\tau}_{\hat{4}1} - \tau_{\hat{4}1}}{\tau_{\hat{4}1}} = -\nu_{\hat{4}}$	$1 - (\tilde{\tau}_{\hat{4}1} + \tilde{\tau}_{\hat{4}3} + \tilde{\tau}_{\hat{4}4})$	$\frac{\tilde{\tau}_{\hat{4}3} - \tau_{\hat{4}3}}{\tau_{\hat{4}3}} = -\nu_{\hat{4}}$	$\frac{\tilde{\tau}_{\hat{4}4} - \tau_{\hat{4}4}}{\tau_{\hat{4}4}} = -\nu_{\hat{4}}$

Abbreviations: SHC = Sexual Health Centres

Footnote. Calculations of the intervention transition probabilities after a positive test result ($\tilde{\lambda}_{\hat{\imath}i}$) are the same as the calculation of the intervention transition probabilities after a negative test result ($\tilde{\delta}_{\hat{\imath}j}$), but with different original transition probabilities.

[a] Initial subgroup

[b] Subgroup an individual can move to

Table S5. Intervention effect in each subgroup for different intervention scenarios

**Intervention scenarios**	Low-impulsivity $(\nu_{\hat{1}})$	Condom-using $(\nu_{\hat{2}})$	Insecure $(\nu_{\hat{3}})$	Confident $(\nu_{\hat{4}})$
**Condom promotion SHC**				
Non-differential intervention effect	0.1	0.1	0.1	0.1
Differential intervention effect*	0.1	0.05	0.2	0.15
UA partially differential intervention effect	0.05	0.05	0.2	0.2
UA alternate differential intervention effect	0.1	0.05	0.15	0.2
**Impulsiveness intervention SHC**				
Non-differential intervention effect	0.1	0.1	0.1	0.1
Differential intervention effect*	0.05	0.1	0.2	0.15
UA partially differential intervention effect	0.05	0.05	0.2	0.2
UA alternate differential intervention effect	0.05	0.1	0.15	0.2
**Condom promotion campaign**				
Non-differential intervention effect	0.05	0.05	0.05	0.05
Differential intervention effect*	0.05	0.025	0.1	0.075
UA partially differential intervention effect	0.025	0.025	0.1	0.1
UA alternate differential intervention effect	0.05	0.025	0.075	0.1

* Differential intervention effect = intervention tailored to subgroup-specific characteristics
Abbreviations: UA=Uncertainty Analysis

S2.2. Calculation of the reduction in chlamydia prevalence

Figure S3 shows how the reduction in total chlamydia prevalence, and the reduction in chlamydia prevalence in each subgroup, was calculated after the introduction of the intervention scenario. In this example, the reduction in total chlamydia prevalence is shown, but the method also applies to calculating the relative reduction for each subgroup. The reduction in this example was obtained by calculating the difference in prevalence between year 0 (a) and year 5 (b) divided by the prevalence in year 0 (a). For example, the reduction in the total prevalence of chlamydia five years after the introduction of intervention scenario 1 in the total population was (10.7% - 13.0%)/13.0% = -0.18. Thus, the estimated chlamydia prevalence in the total population was 17% lower than before the introduction of the intervention scenario.

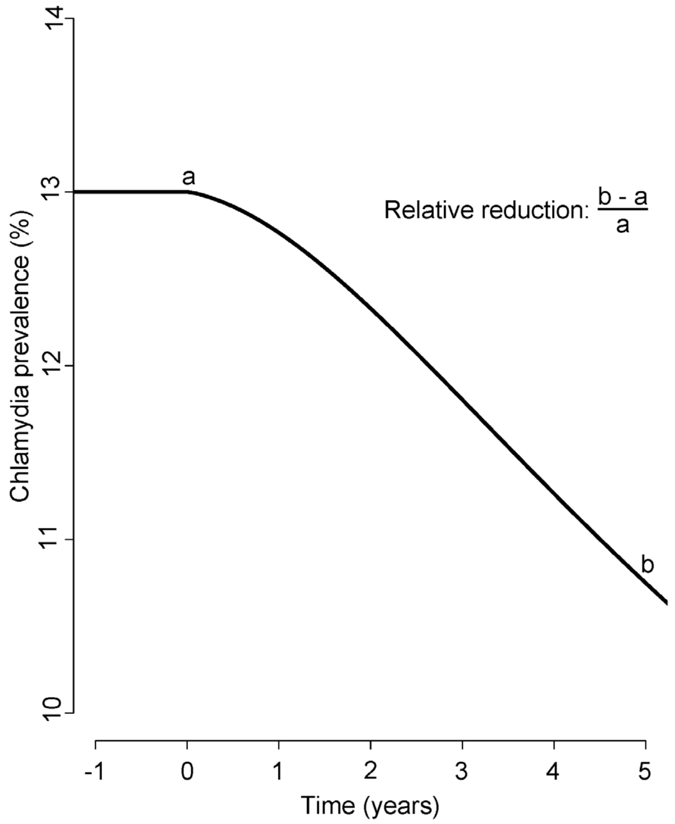

Figure S3. Example of the calculation of the relative reduction in the total prevalence of chlamydia five years after the introduction of an intervention.

S3. Additional results

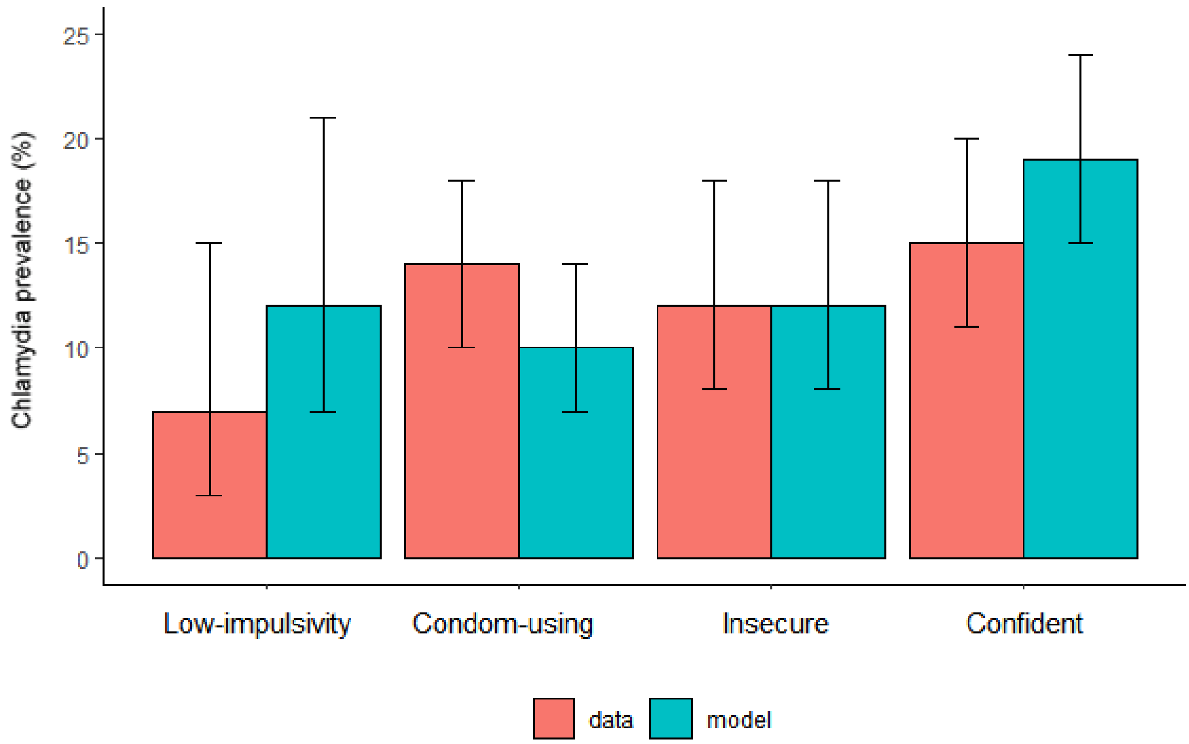

Figure S4. Model fit of chlamydia prevalence to chlamydia positivity rates in the iMPaCT data in each subgroup, including 95% confidence intervals.

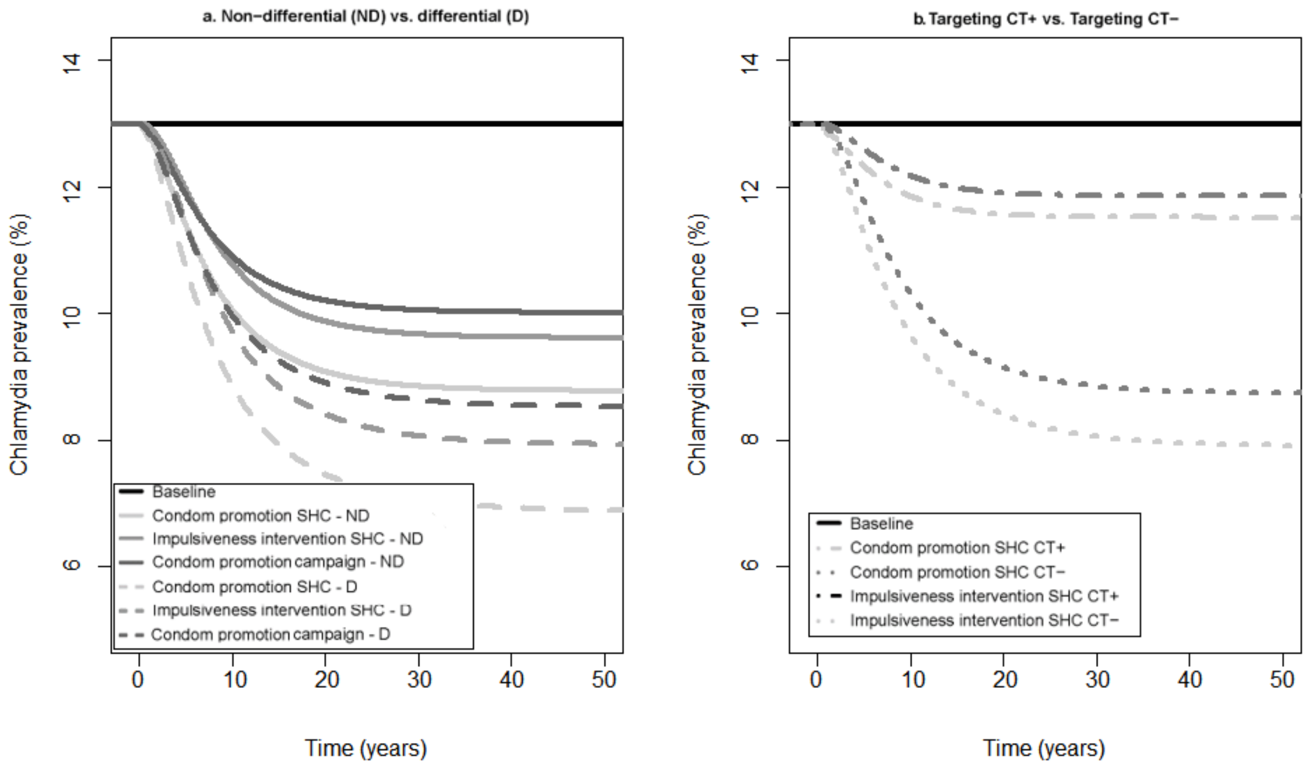

Figure S5. In the left panel (a), the impact of introducing condom promotion at SHC, an impulsiveness intervention at SHC, and condom promotion campaign, assuming a non-differential (ND) intervention effect (solid lines), and a differential (D) intervention effect (dashed lines) on overall chlamydia prevalence for fifty consecutive years is shown. On the right panel (b), the impact of introducing condom promotion at SHC, and an impulsiveness intervention at SHC with differential intervention effects, assuming that only individuals who were diagnosed with chlamydia were targeted (CT+, dash-dotted lines), and that only individuals who tested chlamydia negative were targeted (CT-, dotted lines), on overall chlamydia prevalence for fifty consecutive years is shown. Note that the y-axis starts at 5% to better visualize the differences between the interventions.

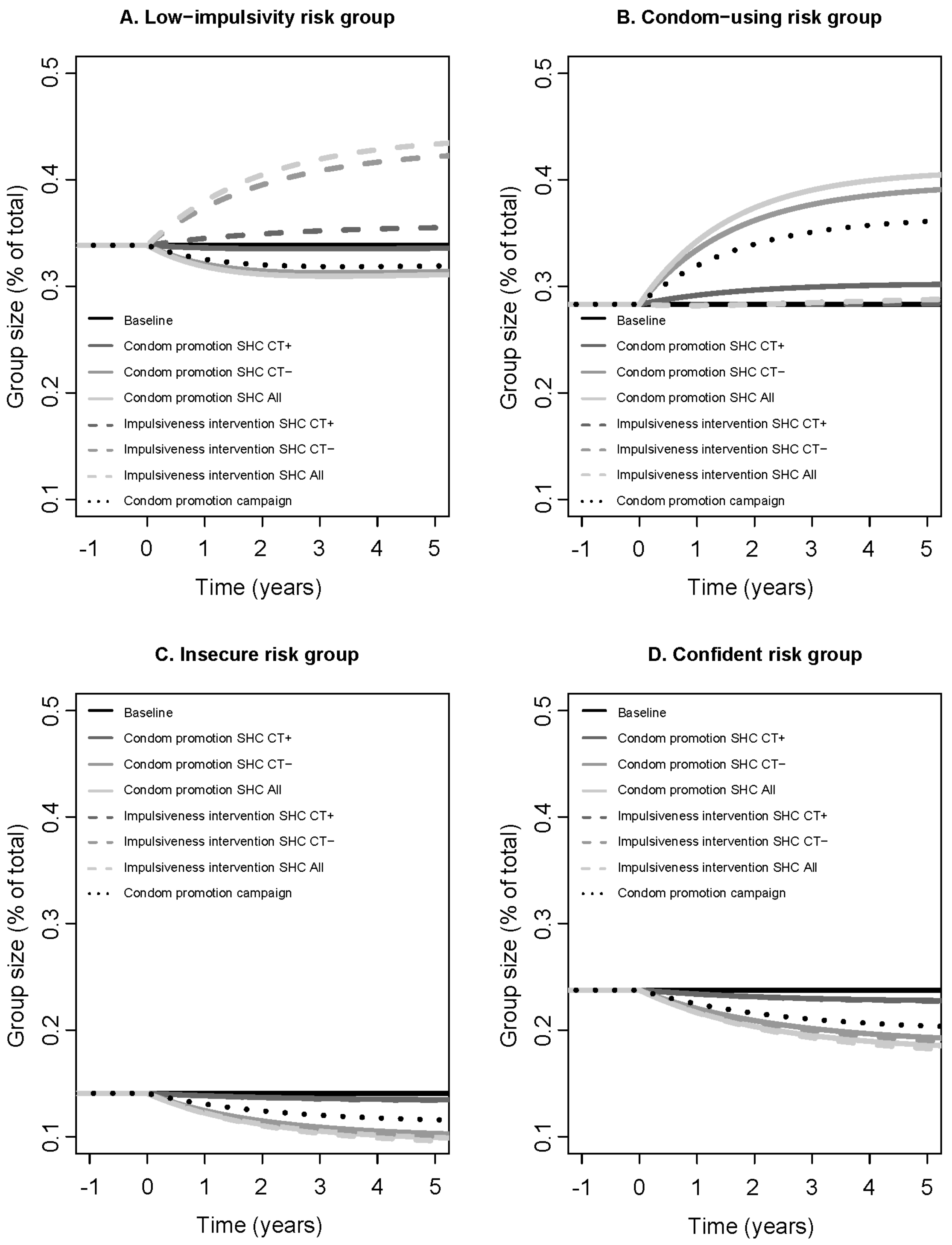

Figure S6. The impact of introducing condom promotion at SHC, an impulsiveness intervention at SHC, and condom promotion campaign, assuming a differential intervention effect on the group sizes of the low-impulsivity subgroup (A), condom-using subgroup (B), insecure subgroup (C), and the confident subgroup (D). The subgroup size is shown for five consecutive years after the introduction of the interventions.

References

1 van Wees, D. A. *et al.* Longitudinal patterns of STI risk based on psychological characteristics and sexual behavior in heterosexual STI clinic visitors. *Sex Transm Dis* **47**, 171-176, doi:10.1097/OLQ.0000000000001110 (2020).

2 Althaus, C. L., Heijne, J. C. M., Roellin, A. & Low, N. Transmission dynamics of Chlamydia trachomatis affect the impact of screening programmes. *Epidemics* **2**, 123-131, doi:10.1016/j.epidem.2010.04.002 (2010).

3 Price, M. J. *et al.* Mixture-of-exponentials models to explain heterogeneity in studies of the duration of Chlamydia trachomatis infection. *Stat Med* **32**, 1547-1560, doi:10.1002/sim.5603 (2013).

4 van Wees, D. A. *et al.* The impact of STI test results and face-to-face consultations on subsequent behaviour and psychological characteristics. *Submitted* (2020).

5 Hartney, T., Baraitser, P. & Nardone, A. Self-Reported Impact of Chlamydia Testing on Subsequent Behavior: Results of an Online Survey of Young Adults in England. *Sex Transm Dis* **42**, 486-491, doi:10.1097/OLQ.0000000000000323 (2015).
